# Uncovering a novel treatment strategy: sodium butyrate overcomes cisplatin resistance in the oral squamous cell carcinoma by inducing ferroptosis

**DOI:** 10.1186/s13046-026-03663-0

**Published:** 2026-02-16

**Authors:** Bing Wang, Wei Li, Yujia Bai, Zhangci Su, Qingwen Zeng, Chao Lv, Qinchao Hu, Bin Cheng, Xiaoan Tao

**Affiliations:** 1 Hospital of Stomatology, Guanghua School of Stomatology, Sun Yat-sen University, No.56, Lingyuan Xi Road, Guangzhou, Guangdong 510055 China; 2 Guangdong Provincial Key Laboratory of Stomatology, Guangzhou, China

**Keywords:** Oral squamous cell carcinoma, Cisplatin resistance, Sodium butyrate, Ferroptosis

## Abstract

**Background:**

Chemoresistance to platinum-based agents like cisplatin is a major therapeutic challenge in advanced oral squamous cell carcinoma (OSCC), with 70–80% of recurrent cases developing resistance that severely compromises clinical outcomes. The mechanism of cisplatin resistance still remains unclear and requires further investigation. This study investigated ferroptosis suppression as a mechanism underlying this resistance and explored the therapeutic potential of the histone deacetylase (HDAC) inhibitor sodium butyrate (NaB).

**Methods:**

Cisplatin-resistant OSCC cells (CAL27/CDDP) and parental cells (CAL27) were used to assess ferroptosis levels and resistance mechanisms. The effects of NaB on reversing cisplatin resistance and inhibiting malignant behaviors (proliferation, migration, invasion) were evaluated in vitro. Mechanistic studies, including identification of key regulators and epigenetic modifications were conducted. The therapeutic effect was further validated in vivo using xenograft tumor models treated with NaB and cisplatin.

**Results:**

CAL27/CDDP exhibited significant ferroptosis suppression compared to CAL27. NaB effectively reversed cisplatin resistance and inhibited malignant behaviors in both cell lines. Mechanistic exploration revealed that NaB enhanced acetylation at the promoter region of early growth response protein 1 (EGR1) through HDAC9 inhibition, elevating its transcriptional activity. EGR1 functioned as a transcription factor to upregulate cytochrome P450 oxidoreductase (POR) expression, potentiating ferroptosis execution. In vivo experiments further confirmed the therapeutic relevance of this HDAC9/EGR1/POR signaling pathway, as NaB administration significantly sensitized xenograft tumors to cisplatin treatment.

**Conclusions:**

These findings position NaB as a promising epigenetic modulator for overcoming cisplatin resistance in OSCC models, with immediate clinical implications. The identified pathway offers a redox biology-based therapeutic strategy that could be extended to other chemotherapy-resistant solid tumors, potentially revolutionizing the treatment paradigm for recurrent malignancies.

**Supplementary Information:**

The online version contains supplementary material available at 10.1186/s13046-026-03663-0.

## Introduction

Cisplatin (CDDP) is one of the most effective anticancer drugs and has been used in the treatment of diverse solid malignancies, including head and neck cancer [1], lung cancer [2], testicular cancer [3], bladder cancer [4] and gastric cancer [5]. Cisplatin-based chemotherapy is indispensable in current neoadjuvant therapy, complemented by the importance of targeted and immune-based therapies [6]. A meta-analysis of 10 articles, encompassing 1317 samples, demonstrated that the frequency of cisplatin resistance was 33% [7].

Cisplatin exerts an anticancer effect through multiple mechanisms, one of which is the activation of the DNA damage response and following induction of cell death [8]. However, the mechanism of cisplatin resistance is quite complex and involves many aspects, such as alternations in drug transport and metabolism, increased DNA repair, cell cycle regulation, inhibition of programmed cell death and so on [9, 10]. Moreover, epigenetic modification has also been recognized as one of the resistance mechanisms, including alterations in DNA methylation patterns, noncoding RNA regulation, N6-adenosine methylation (m6A) modification of mRNA, and posttranslational modifications [8]. Therefore, understanding the mechanism of cisplatin resistance and overcoming these clinically relevant issues are critical challenges in the management of patients with advanced solid tumor.

Ferroptosis, an iron-dependent programmed cell death, is mediated by cellular lipid peroxides accumulation to initiate membrane oxidative damage [11]. Ferroptosis escape mediated by certain oncogenes or oncogenic signaling pathways can facilitate tumor progression and resistance to chemotherapy. It has been shown that ferroptosis inhibition is one of the major concerns in chemoresistance [12]. Reactive oxygen species (ROS) produced by cisplatin which can directly trigger DNA damage may be neutralized by reductive metabolites [13]. A spontaneously occurring temperature increase in solid tumors confers protection to lipid peroxidation and contributes to ferroptosis evasion and chemotherapy resistance [14]. In addition to metabolites that can affect chemoresistance, the alternation of genes related to ferroptosis is also a crucial reason for chemoresistance [15]. Cytochrome P450 oxidoreductase (POR) as a key mediator of lipid peroxidation, donates electrons to redox partners and enables membrane polyunsaturated phospholipid peroxidation to induce rapid membrane damage and subsequent ferroptosis, which shows giant therapeutic value [16]. Ferroptosis provides a new prospective for the treatment of chemoresistance malignancies and recently garnered great interest in cancer research.

Oral squamous cell carcinoma (OSCC) developing from oral mucosal epithelium is the most common malignancy of the oral cavity [17]. According to data from Global Cancer Observatory in 2022, the age-standardized incidence and mortality rate of OSCC is 5.8 and 2.3 per 100,000 person-years respectively [18]. A large-scale OSCC study in Taiwan indicated that 30% of the patients suffered a relapse following chemotherapy treatment [19]. The mechanism underlying cisplatin resistance in OSCC is similar to other solid tumors [8, 9, 20]. Furthermore, cisplatin-resistant OSCC cells reprogram lipid metabolism to reduce polyunsaturated fatty acid (PUFA)-containing phospholipids, which are essential substrates for lipid peroxidation during ferroptosis [21].

Short chain fatty acids (SCFA), especially butyric acid, as metabolites of oral and intestinal microbiota, are important energy sources and signaling molecules of mucosal epithelium. The ionized form of butyric acid, sodium butyrate (NaB), exhibits potent antitumor activity especially malignancy of epithelial origin [22]. NaB is a natural histone deacetylase (HDAC) inhibitor which affects essential regulation of gene transcription various, protein-protein interactions (PPIs) and different posttranslational modifications to be involved in several physiological processes in the pathogenesis of numerous diseases [23]. However, the role of NaB in mitigating resistance to cisplatin-based chemotherapy remains uncertain.

In this study, we identified a promising epigenetic-based therapeutic approach to overcome cisplatin resistance in OSCC. Through systematic comparative analysis between cisplatin-sensitive and resistant OSCC cell lines, we discovered that ferroptosis resistance represents a critical survival mechanism in chemoresistant cells. Significantly, NaB functioning as a HDAC inhibitor, was demonstrated to overcome this resistance through a novel redox-dependent mechanism. These findings not only elucidate a previously unrecognized chemoresistance pathway but also position NaB as a dual-function agent capable of simultaneously reversing drug resistance and modulating redox homeostasis, highlighting its potential for clinical translation for recurrent OSCC management.

## Materials and methods

### Cell culture

The human oral squamous cell carcinoma (OSCC) cell lines CAL27, CAL33, HSC3, SCC15 were procured from the American Type Culture Collection (ATCC, MD, USA). CAL27/CDDP which is resistant to cisplatin was provided by Jinsong Li (the Second Affiliated Hospital of Sun Yat-sen University). According to the previous study, the stable cisplatin-resistant line CAL27/CDDP was established by treating cisplatin-sensitive cell lines CAL27 with gradually increasing doses of cisplatin from 0.1 µM to 10 µM in cell culture medium until the survival cells exhibited a normal growth pattern [24, 25]. CAL27, CAL33, HSC3, SCC15 and CAL27/CDDP were cultured in Dulbecco’s modified Eagle medium (DMEM; Gibco, MD, USA) supplemented with 10% fetal bovine serum (FBS; Gibco, MD, USA), and CAL27/CDDP was additionally supplemented with 0.5 µM cisplatin (CDDP, BD121788, Bidepharm, Shanghai, China). All cells were incubated in a humidified atmosphere containing 5% CO_2_ at 37℃.

### CCK8 viability assay

CAL27, CAL33, HSC3, SCC15 and CAL27/CDDP cells were seeded at a density of 8000 cells/ well in 96-well plates. After the cells adhered overnight, the control group and the experimental group were treated with basal medium supplemented with varying concentrations of cisplatin and sodium butyrate (NaB, B5887, Sigma-Aldrich, Germany), and the cells were cultured in the corresponding conditions. After 24 h, Cell Counting Kit-8 (CCK8, CK04, DOJINDO, Japan) reagent was added to each well according to the manufacturer’s instruction to assess cell proliferation. The absorbance at 450 nm was subsequently measured using a microplate reader. The cell viability of CAL27, CAL33, HSC3, SCC15 and CAL27/CDDP treated with a combination of the ferroptosis inducer erastin (10 µM, HY-15763, MCE, Shanghai, China) or the inhibitor ferrostatin-1 (Fer-1, 10 µM, HY-100579, MCE, Shanghai, China) and cisplatin was detected using the same method. Similarly, the same method was used to evaluate the effects of NaB (5 mM), as well as the combined effects of NaB and Fer-1 (10 µM), on the cellular activities of CAL27, CAL33, HSC3, SCC15 and CAL27/CDDP at various time points. CDDP and NaB were added to 96-well plates at a range of concentration ratios to establish a dose-response matrix. Synergistic effects were quantified using the Zero Interaction Potency (ZIP) model via the SynergyFinder web application (https://synergyfinder.aittokallio.group). A ZIP synergy score greater than 10 was used as the threshold for defining synergy.

### ROS detection

CAL27 and CAL27/CDDP cells were seeded at a density of 3 × 10⁵ cells/ well in 6-well plates. When the cell density reached approximately 60–70%, the cells were treated and then stained with dihydroethidium (DHE) Kit (C1300, Applygen, Beijing, China) at 37℃ for 30 min. Finally, intracellular ROS were detected by flow cytometry.

### Lipid ROS detection

CAL27 and CAL27/CDDP cells were seeded at a density of 3 × 10⁵ cells per well in 6-well plates. When the cell density reached approximately 60–70%, the cells were treated and then stained with BODIOY C11(D3861, Thermo Fisher, MD, USA) at 37℃ for 20 min. Finally, Lipid ROS were detected by flow cytometry. The cells seeded on sterile cover glass were observed under a fluorescence microscope, and the staining procedure was the same as above.

### Fe^2+^ detection

CAL27 and CAL27/CDDP cells were seeded into 24-well plates at a density of 1 × 10⁵ cells/well. Intracellular Fe²⁺ levels were detected using the FerroOrange probe (F374, DOJINDO, Japan), as follows: culture medium was aspirated and discarded, followed by washing with HBSS. The FerroOrange probe was diluted to 1 µM in HBSS, and the working solution was added to the cells. After incubation at 37 °C for 30 min, fluorescence microscopy was performed for observation.

### Measurement 4-hydroxynonenal (4-HNE) by ELISA

4-HNE levels were measured using a commercial ELISA kit (E-EL-0128, Elabscience, Wuhan, China) following the manufacturer’s instructions. Standard wells, blank wells, and sample wells were set up and loaded with standard working solution, standard diluent, and cell supernatant, respectively. After adding 50 µL of biotinylated antibody working solution to each well, plates were incubated at 37 °C for 45 min followed by three washes. Then, 100 µL of HRP enzyme conjugate working solution was added and incubated at 37 °C for 30 min with five subsequent washes. For detection, 90 µL of substrate solution was added and incubated at 37 °C in the dark for 15 min before terminating the reaction with 50 µL of stop solution. Finally, absorbance was measured at 450 nm using a microplate reader.

### Transmission eletron microscopy (TEM)

TEM was used to observe the ultrastructure of the OSCC cells. CAL27/CDDP and CAL27 cells in logarithmic growth phase (1 × 10^7^ cells) were collected by trypsin digestion and centrifugation into clusters and rapidly placed in precooled 0.1 M phosphate buffer (PB) to prepare 2.5% glutaraldehyde fixative for 4 h. The cells were washed with PB three times and then transferred into 1% osmic acid solution and fixed overnight at 4℃. Then, cells were dehydrated by gradient ethanol and embedded at 60℃ for 48 h. Finally, the samples were cut into ultrathin sections and observed under TEM (JEOL, Janpan).

### RNA extraction and quantitative real-time PCR (qPCR)

Total RNA was isolated from CAL27 and CAL27/CDDP cells using a commercial RNA extraction kit (RC102, Vazyme, Nanjing, China). 300 ng mRNA was reverse transcribed into cDNA using HiScript All-in-one Ultra RT SuperMix (R333, Vazyme, Nanjing, China). Quantitative PCR was performed with ChamQ Universal SYBR qPCR Master Mix (Q711, Vazyme, Nanjing, China) according to the manufacturer’s protocol. Each 20 µL reaction mixture contained 1 µL cDNA, 10 µL SYBR Green Master Mix, 0.4 µL each of forward and reverse primers, and 8.2 µL nuclease-free water. Amplification was carried out on a QuantStudio7 FLEX Real-Time PCR System under the following conditions: initial denaturation at 95 °C for 30 s, followed by 40 cycles of 95 °C for 10 s and 60 °C for 30 s, with subsequent melt curve analysis. β-actin served as the endogenous control, and relative gene expression was calculated using the 2-ΔΔCT method. The primers used in this study, purchased from BGI Tech Solutions (BEIJING LIUHE, Beijing, China) were listed in Table S1 (Supporting information).

### Western blot

CAL27 cells and CAL27/CDDP cells in the logarithmic growth phase were collected, washed three times with PBS and lysed with RIPA buffer (CW2333, CoWin Biotech, Jiangsu, China) on ice for 20 min. After centrifugation at 4 ° C for 20 min, the supernatant of lysates was collected. Protein concentration was quantified using the bicinchoninic acid (BCA) kit (CW0014, CoWin Biotech, Jiangsu, China), followed by addition of SDS-PAGE loading buffer (CW0028, CoWin Biotech, Jiangsu, China) to mix thoroughly, denaturation at 95 ℃ for 10 min, and place on ice. 20 µg Proteins were separated by SDS-PAGE and transferred to polyvinylidene fluoride (PVDF) membranes. The membranes were blocked at room temperature for 1 h, followed by overnight incubation in primary antibody solution at 4 ° C. After washing three times with triethanolamine buffered saline solution (TBS), the membranes were incubated in secondary antibodies which were anti-rabbit or anti-mouse HRP-conjugated antibodies (1:10000, EMAR, Beijing, China) for 1 h at room temperature. After washing three times with TBS, the values of immunoreactive bands were detected by chemiluminescent HRP substrate (WBULS0100, Millipore, MA, USA) and quantified by Image J software. The antibodies applied in this study were listed in Table S2 (Supporting information).

### EdU proliferation assay

Cell proliferation was assessed using the EdU assay. EdU staining was carried out in accordance with the manufacturer’s guidelines (C0078, Beyotime Biotechnology, Shanghai, China). CAL27 and CAL27/CDDP cells (3 × 10^4^ cells *per* well) in the logarithmic growth phase were seeded in 24-well plates. CAL27 and CAL27/CDDP cells were treated with 5 mM NaB as well as 5 µM CDDP for 24 h. The EdU (10 mM) solution was diluted to a concentration of 10 µM with complete medium, and equal volumes were added to 24-well plates and incubated for 2 h. At the same time, a negative control group without EdU labeling treatment was set. Cells were fixed and permeabilized before washing twice. Subsequently, EdU staining working solution and Hoechst staining working solution were added to each well in turn, and appropriate detection channels were selected using fluorescence microscopy after incubation at room temperature. Proliferating cells marked with EdU emitted a vibrant red fluorescence, while all viable cells stained with Hoechst 33,342 exhibited a brilliant blue fluorescence. The relative number of EdU-labeled cells was determined by dividing the number of EdU-positive cells by the total cell count in each field of view.

### Cell migration and invasion assay

To examine the migration ability of the cells, a wound healing assay was used. In brief, CAL27 and CAL27/CDDP cells in logarithmic growth phase were seeded in 6-well plates at 3 × 10^5^ cells/well, and the cells were allowed to confluence to 80% for further treatment. A sterile 200 µL yellow pipette tip was used for the scratch vertically. Cells were washed with PBS to remove the scratched cells. Pictures were taken under a microscope and recorded for 0 h. After 24 h of drug treatment, the cells were photographed under a microscope and recorded for 24 h. The migration rate was calculated using Image J software.

To detect cell invasion potential, a transwell invasion assay (8-µm pore size; Corning, NY, USA) was performed. In brief, matrigel was diluted with serum-free medium and uniformly spread on the upper chamber (transwell chambers), and then incubated at 37 °C for 3 h. CAL27 and CAL27/CDDP cells in logarithmic growth phase were seeded at a density of 1 × 10^5^ cells *per* chamber. The cells were resuspended in serum-free medium containing 5 mM NaB and 5 µM CDDP. In the lower chamber, high-glucose DMEM medium containing 10% FBS was added and incubated for 24 h. After the chamber was removed carefully, the Matrigel and cells on the upper surface of the membranes were gently wiped off with cotton swabs and fixed with 4% paraformaldehyde for 20 min. After washing with PBS for 3 times, 0.5% crystal violet (G1062, Solarbio, Beijing, China) staining was applied for 15 min and photographs were taken under microscope to detect the count of invading the bottom surface of the membrane.

### Lentivirus production and cell transfection

GFP fusion plasmids were purchased from GeneCopoeiaTM (GeneCopoeiaTM, MD, USA). Lentiviral vector production was performed with the Lenti-Pac™ HIV Packaging kit (GeneCopoeiaTM, MD, USA). HEK-293 cells were transfected with the packaging mixture and GFP plasmid vector, respectively. Conditioned medium from transfected HEK-293 cells was collected 48 h later and used to infect OSCC cell lines. OSCC cells were cultured in 2.5 µg /mL purinomycin (58-58-2, BioFroxx, Germany) to screen for stable expression of target plasmids. The shPOR sequences were list in Table S3 (Supporting information).

### Dual-luciferase reporter assay

Various wild-type or mutated POR promoter fragments were subcloned and inserted into the pGL3-basic vector to construct pGL3-POR-WT-Luc, pGL3-POR-MutA-Luc, pGL3-POR-MutB-Luc, and pGL3-POR-MutC-Luc (Kidan Bio, Guangzhou, China). EGR1 fragment were subcloned and inserted into the pcDNA3.1(+) vector to construct pcDNA3.1(+)-EGR1 (Kidan Bio, Guangzhou, China). CAL27/CDDP cells were transfected with these constructs and pRL-TK plasmids. The pRL-TK plasmid is designed for Renilla luciferase reporter assays in mammalian cells. It serves as an internal control in dual-luciferase assays (with firefly luciferase reporters) to normalize transfection efficiency. Luciferase activity was detected via a dual luciferase reporter assay kit (RG027, Beyotime Biotechnology, Shanghai, China) to determine firefly/renilla luciferase activity. The sequences of POR promoter were listed in Table S4 (Supporting information).

### Transient transfection of SiRNA

To determine the function of target proteins in CAL27/CDDP cells, small interfering RNA (siRNA) oligonucleotide (Kidan Bio, Guangzhou, China) was applied. Lipo8000™ Transfection Reagent (C0533, Beyotime Biotechnology, Shanghai, China) was used to promote the transfection of siRNA oligonucleotides into the targeted cells according to manufacturer’s guidelines. The EGR1 and HDAC9 siRNAs sequences were list in Table S3 (Supporting information).

### Pan-HDAC activity assay

CAL27/CDDP cells in logarithmic growth phase were collected and lysed. According to the manufacturer’s instructions (13601, AAT Bioquest, CA, USA), the samples were incubated with HDAC inhibitor and HDAC Green substrate working solution successively at 37 °C. The absorbance at 490 nm was measured using a microplate reader.

### Chromatin Immunoprecipitation (ChIP) -qPCR assay

ChIP assay was performed using Pierce™ Magnetic ChIP Kit (26157, Thermo Fisher, MD, USA). Briefly, CAL27/CDDP cells were fixed with 1% formaldehyde solution for 10 min at room temperature and terminated with 0.125 M glycine for 5 min. Fixed cells were lysed on ice for 10 min and digest with MNase Digestion Buffer at 37 °C for 15 min. The chromatin was then sonicated on ice into 500 bp fragments. DNA obtained after sonication was used as input control. Immunoprecipitations were performed with Anti-Histone H3K27ac antibody or normal rabbit IgG antibody or Anti-RNA Polymerase II Antibody. After overnight incubation at 4 °C, DNA was pulled down using ChIP grade protein A/G magnetic beads. Samples were then reverse crosslink and treatment with Proteinase K and finally purified for subsequent qPCR analyses and agarose gel electrophoresis. Primers were listed in Table S1 (Supporting information).

### Animal model

Balb/c nude mice were purchased from GemPharmatech (Jiangsu, China). All mice were housed in specific pathogen-free conditions at the Laboratory Animal Center of Sun Yat-sen University. The Laboratory Animal Care and Use Committee of Sun Yat-sen University has approved this part of the study (SYSU-IACUC-2025-000490). CAL27/CDDP cells in logarithmic growth phase were collected, and 5 × 10^5^ cells/site were subcutaneously injected into the axilla to establish the nude mouse xenograft models. Drug treatment was started 1 week later. Cisplatin (2 mg/kg) and Fer-1 (5 mg/kg) were administered intraperitoneally once every 3 days. NaB was dissolved with distilled water at a concentration of 200 mM, administered by drinking water, and changed every 3 days. Body weight and tumor growth were monitored, and tumor length and width were measured.

### Histological hematoxylin-eosin (HE) staining and immunohistochemistry (IHC)

For HE staining, samples were fixed in 4% paraformaldehyde (P1110, Solarbio, Beijing, China), subsequently dehydrated and embedded in paraffin. The embedded tissue samples were prepared into paraffin sections with a thickness of 4 μm and stained with hematoxylin and eosin solutions (G1080; G1100, Solarbio, Beijing, China).

IHC was carried out according to the manufacture’s guidelines of IHC Kit (GK600710, GeneTech, Shanghai, China). Briefly, the tissue sections were deparaffinized twice in xylene, sequentially rehydrated in gradient ethanol (100,95,85, and 75% ethanol), and then washed three times with phosphate-buffered saline (PBS, pH 7.4). According to the instructions of the antibody and the preliminary experiment, the appropriate pH antigen recovery buffer was selected: sodium citrate buffer (pH 6.0) or Tris-EDTA buffer (pH 9.0). The microwave oven was turned to high heat and heated above 95℃ for 15 min, and then naturally cooled to room temperature. The endogenous peroxidase activity was blocked for 15 min by solution A of the secondary antibody kit, namely non-specific staining blocker. The sections were rinsing with PBS three times. Goat serum was applied to block nonspecific sites for 30 min. Tissue sections were subsequently incubated with diluted primary antibodies overnight at 4 °C. After rinsing three times with PBS, the secondary antibody of the corresponding species (reagent B solution) was added and incubated for 30 min. The tissue sections were stained with the diaminobenzidine (DAB), immediately observed under microscope and timed. All samples were stained at the same time. Then the tissue sections were counterstained with hematoxylin, dehydrated and sealed, and scanned by an automatic slice scanner for observation and analysis. The antibodies applied in this assay were listed in Table S2 (Supporting information).

### Assessment of liver and kidney function in animals

Liver function was assessed by measuring serum Alanine aminotransferase (ALT) and Aspartate aminotransferase (AST). According to the instructions of Alanine aminotransferase assay kit and Aspartate aminotransferase assay kit (C009-2-1; C010-2-1, Nanjing Jiancheng Bioengineering Institute, Nanjing, China), the serum of nude mice in each group was collected, and the OD values of each well at 505 nm and 510 nm were measured by microplate reader after incubation with reagent 1 and reagent 2 in turn.

Kidney function was assessed by measuring serum Creatinine (Cr) and serum Urea nitrogen (BUN). Cr was detected according to the instructions of the Creatinine Assay kit (C013-2-1, Nanjing Jiancheng Bioengineering Institute, Nanjing, China) as follows: set blank wells, test wells and control wells, and add distilled water, animal serum and standard substance, respectively. The enzyme solution A was added to each well and incubated at 37 ° C for 5 min. The OD values at 546 nm were measured by microplate reader and recorded as A1. Subsequently, the enzyme solution B was added to each well and incubated at 37 °C for 5 min. The OD values at 546 nm were measured by microplate reader and recorded as A2. According to the standard concentration, the Cr concentration of each sample was calculated.

BUN was detected according to the instructions of the Urea assay kit (C011-2-1, Nanjing Jiancheng Bioengineering Institute, Nanjing, China) as follows: set blank tubes, test tubes, and control tubes to add distilled water, animal serum, and standard substance, respectively. Buffer enzyme solution was added to each tube and the reaction was carried out in a water bath at 37 °C for 10 min. Subsequently, phenol chromophoric agent and alkaline sodium hypochlorite solution was successively added to each tube, and the reaction was carried out in a water bath at 37 °C for 10 min. The OD value at a 640 nm was measured by microplate reader. According to the standard concentration, the BUN concentration of each sample was calculated.

### Statistical analysis

Normally distributed data were presented as mean ± standard deviation (Mean ± SD) from replicated experiments. GraphPad Prism 9 (GraphPad Software, CA, USA) was used for statistical analysis and visualization of the results. Two independent samples Student’s t test was used to compare the quantitative data between the two groups. One-way analysis of Variance (ANOVA) was used to identify the differences among multiple groups. *P* < 0.05 was considered statistically significant, and the levels of significance were further defined at level of * *P* < 0.05, ** *P* < 0.01, *** *P* < 0.001.

## Results

### Cisplatin resistance cell line CAL27/CDDP is resistant to ferroptosis

**Fig. 1:**
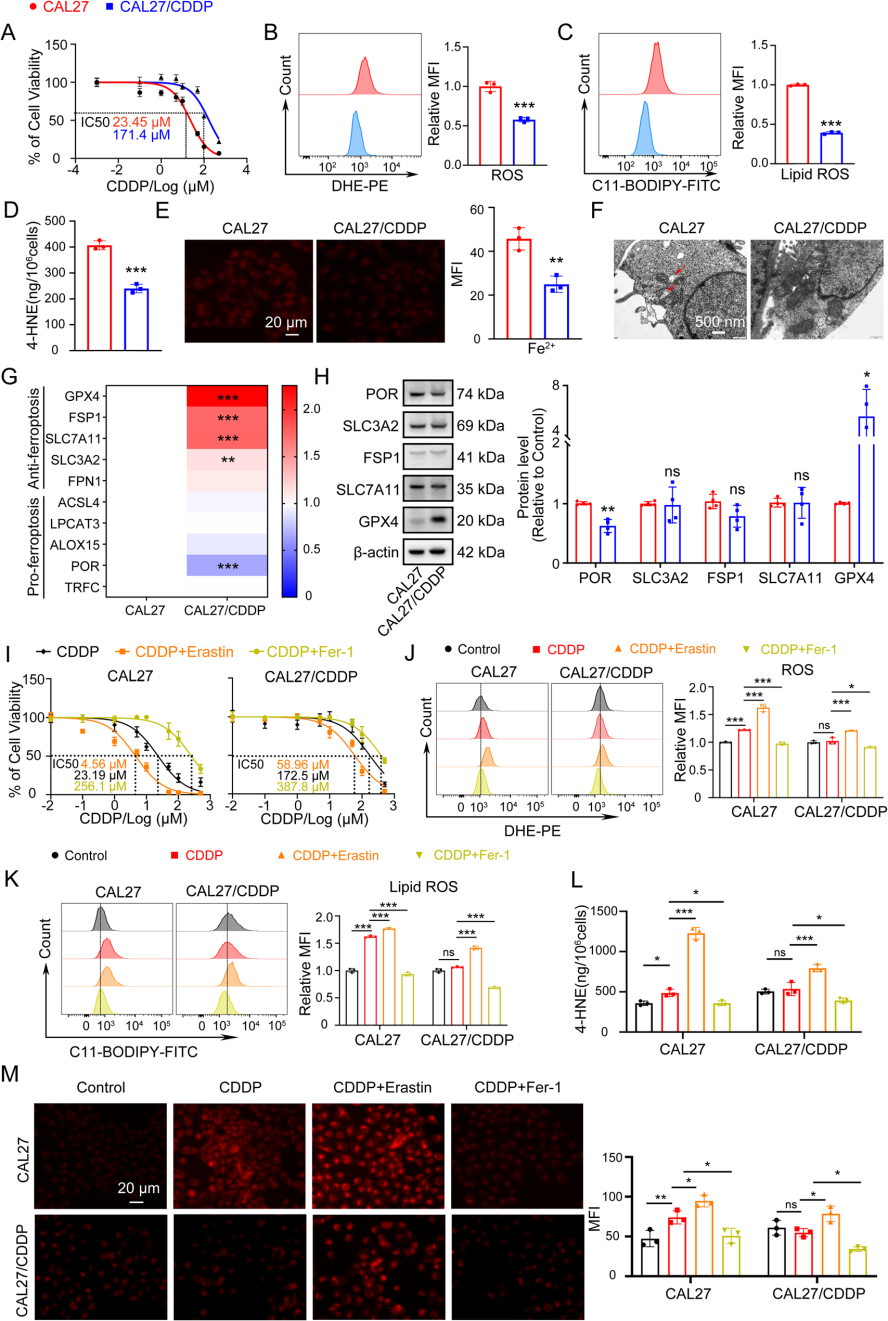
Cisplatin resistance cell line CAL27/CDDP is resistant to ferroptosis. **A** The CCK8 assay was used to determine the IC_50_ of cisplatin in CAL27 and CAL27/CDDP cells. IC_50_, half-maximal inhibitory concentration. **B** The cellular ROS level in CAL27 and CAL27/CDDP cells was detected by using DHE fluorescent probe, which emitted red fluorescence with an excitation maximum at 518 nm and an emission maximum at 610 nm when oxidized. The left panel was representative flow cytometry images and the right panel was quantitative analysis of MFI. ROS, reactive oxygen species. DHE, dihydroethidium. MFI, mean fluorescence intensity. ***, *P* < 0.001. **C** The intracellular lipid ROS level in CAL27 and CAL27/CDDP cells was detected by using C11-BODIPY probe. And when C11 was oxidized, the excitation and emission maxima were shifted to 488 nm and 510 nm, respectively. The left panel was representative flow cytometry images and the right panel was quantitative analysis of MFI. Lipid ROS, lipid peroxides. ***, *P* < 0.001. **D** 4-HNE in CAL27 and CAL27/CDDP cells was quantified using an ELISA assay. 4-HNE, 4-hydroxy-2-nonenal. ***, *P* < 0.001. **E** The intercellular Fe^2+^ concentration in CAL27 and CAL27/CDDP cells was detected by using FerroOrange probe (red). The left panel is representative fluorescent images and the right panel was quantitative analysis of MFI. Scale bar, 20 μm. **, *P* < 0.01. **F** Representative TEM images showed the mitochondrial ultrastructure of CAL27 and CAL27/CDDP cells. The arrows indicated shrunken, hyper-dense mitochondria with a characteristic absence of cristae in CAL27 cells. TEM, transmission electron microscopy. Scale bar, 500 nm. **G** Heat maps of mRNA expressions of ferroptosis related genes in CAL27 and CAL27/CDDP cells detected by RT-qPCR. **, *P* < 0.01 and ***, *P* < 0.001. **H** The protein expressions of ferroptosis related genes in CAL27 and CAL27/CDDP cells was detected by Western blot. The left panel were representative images and the right panel were quantitative analysis. Western blot results were normalized using β-actin as an internal control. *, *P* < 0.05 and **, *P* < 0.01. **I** The CCK8 assay was used to evaluation the IC_50_ of cisplatin in CAL27 and CAL27/CDDP cells following treated with 10 µM erastin and 10 µM Fer-1. **J** Representative DHE flow cytometry images and quantitative analysis of MFI in CAL27 (left panel) and CAL27/CDDP (right panel) treated with 5 µM CDDP, 10 µM erastin and 10 µM Fer-1 for 24 h. *, *P* < 0.05 and ***, *P* < 0.001. **K** Representative C11-BODIPY flow cytometry images, quantitative analysis of MFI in CAL27 (left panel) and CAL27/CDDP (right panel) treated with 5 µM CDDP, 10 µM erastin and 10 µM Fer-1 for 24 h. ***, *P* < 0.001. **L** 4-HNE was quantified using an ELISA assay in CAL27 and CAL27/CDDP cells treated with 5 µM CDDP, 10 µM erastin and 10 µM Fer-1 for 24 h. *, *P* < 0.05 and ***, *P* < 0.001. **M** Representative FerroOrange fluorescent images and quantitative analysis of MFI in CAL27 and CAL27/CDDP treated with 5 µM CDDP, 10 µM erastin and 10 µM Fer-1 for 24 h. Scale bar, 20 μm. *, *P* < 0.05 and **, *P* < 0.01

In order to confirm CAL27/CDDP resistance to cisplatin (CDDP), CAL27 and CAL27/CDDP cells were treated with gradually increasing concentrations of cisplatin (0.1, 1, 5, 10, 50, 100 and 500 µM) for 24 h, and cell viability was measured by CCK-8 assay. As shown in Fig. 1A, the half maximal inhibitory concentration (IC_50_) of CAL27/CDDP was much higher than CAL27, which showed that CAL27/CDDP had lower sensitivity to cisplatin. Given the relationship between ferroptosis inhibition and chemoresistance [21], we wonder that whether the difference in cisplatin sensitivity between the two cell lines was related to ferroptosis, so markers of ferroptosis were detected by the following assays. We determined a significant reactive oxygen species (ROS) reduction in CAL27/CDDP compared to CAL27 by using dihydroethidium (DHE) fluorescent probe (Fig. 1B). At the same time, C11-BODIPY probe was used to assess the intracellular lipid peroxides (lipid ROS) level in two cell lines. The stronger green fluorescence of CAL27 than CAL27/CDDP indicated that more lipid ROS were present in CAL27 (Fig. 1C). Then, 4-hydroxy-2-nonenal (4-HNE), a cytotoxic byproduct generated during lipid peroxidation, was quantified using an ELISA assay. The analysis revealed markedly elevated 4-HNE levels in CAL27 compared to CAL27/CDDP. (Fig. 1D). Furthermore, we used FerroOrange, a Fe^2+^-specific probe, to detect intercellular Fe^2+^ concentration in two cell lines. The mean fluorescence intensity of Fe^2+^ in CAL27/CDDP was significantly less than CAL27 (Fig. 1E). Mitochondrial morphology is also a crucial feature of ferroptosis [26]. The mitochondrial ultrastructure of CAL27 and CAL27/CDDP was observed via transmission electron microscopy (TEM). Compared to CAL27/CDDP, CAL27 cells exhibited shrunken, hyper-dense mitochondria with a characteristic absence of cristae, as indicated by the arrows (Fig. 1F). Additionally, we compared the expression of some ferroptosis relevant genes in the two cell lines. Compared with CAL27, the expression of pro-ferroptosis genes such as cytochrome P450 oxidoreductase (POR) was lower in CAL27/CDDP, while the expression of anti-ferroptosis genes such as glutathione peroxidase 4 (GPX4) was higher (Fig. 1G, H).

To determine whether CAL27/CDDP cells are protected from ferroptosis, both CAL27 and CAL27/CDDP cell lines were treated with CDDP, in the presence or absence of the specific ferroptosis inhibitor (ferrostatin-1, Fer1) or inducer (Erastin). The CCK-8 assay revealed that Erastin sensitized both CAL27 and CAL27/CDDP cells to cisplatin, as evidenced by a significant decrease in IC₅₀. Conversely, Fer-1 substantially increased their resistance to cisplatin, underscoring the role of ferroptosis in mediating cisplatin sensitivity (Fig. 1I). Specifically, CDDP treatment for 24 h induced a robust ferroptotic response in CAL27 cells, as evidenced by a significant increase in intracellular ROS (Fig. 1J) and lipid ROS (Fig. 1K) levels measured using DHE and C11-BODIPY probe, respectively. Additionally, extracellular 4-HNE levels (Fig. 1L) were markedly elevated, as determined by ELISA, and intracellular Fe²⁺ accumulation (Fig. 1M) was observed via the Ferrorange probe, compared to the control group. The combination of erastin (10 µM) and CDDP exacerbated ferroptosis in CAL27 cells. Notably, co-treatment with Fer-1 (10 µM) almost completely abrogated these effects, reducing lipid ROS, 4-HNE, and Fe²⁺ levels to near-baseline values (Fig. 1J–M). In contrast, CAL27/CDDP cells exhibited a markedly attenuated response to the same CDDP administration. No significant increases in ROS, lipid ROS, or 4-HNE levels were detected, and intracellular Fe²⁺ remained unchanged (Fig. 1J–M). However, combined treatment with erastin (10 µM) and cisplatin effectively triggered substantial accumulation of Fe²⁺, ROS, and lipid ROS, along with increased 4-HNE release, in both the parental and resistant cell lines (Fig. 1J–M). Furthermore, co-incubation with Fer-1 (10 µM) and CDDP significantly downregulated these parameters, compared to the CDDP-only group (Fig. 1J–M). In summary, these data indicated that cisplatin-resistance cell line CAL27/CDDP cells exhibited resistance to ferroptosis.

### NaB reverses cisplatin resistance of CAL27/CDDP

**Fig. 2:**
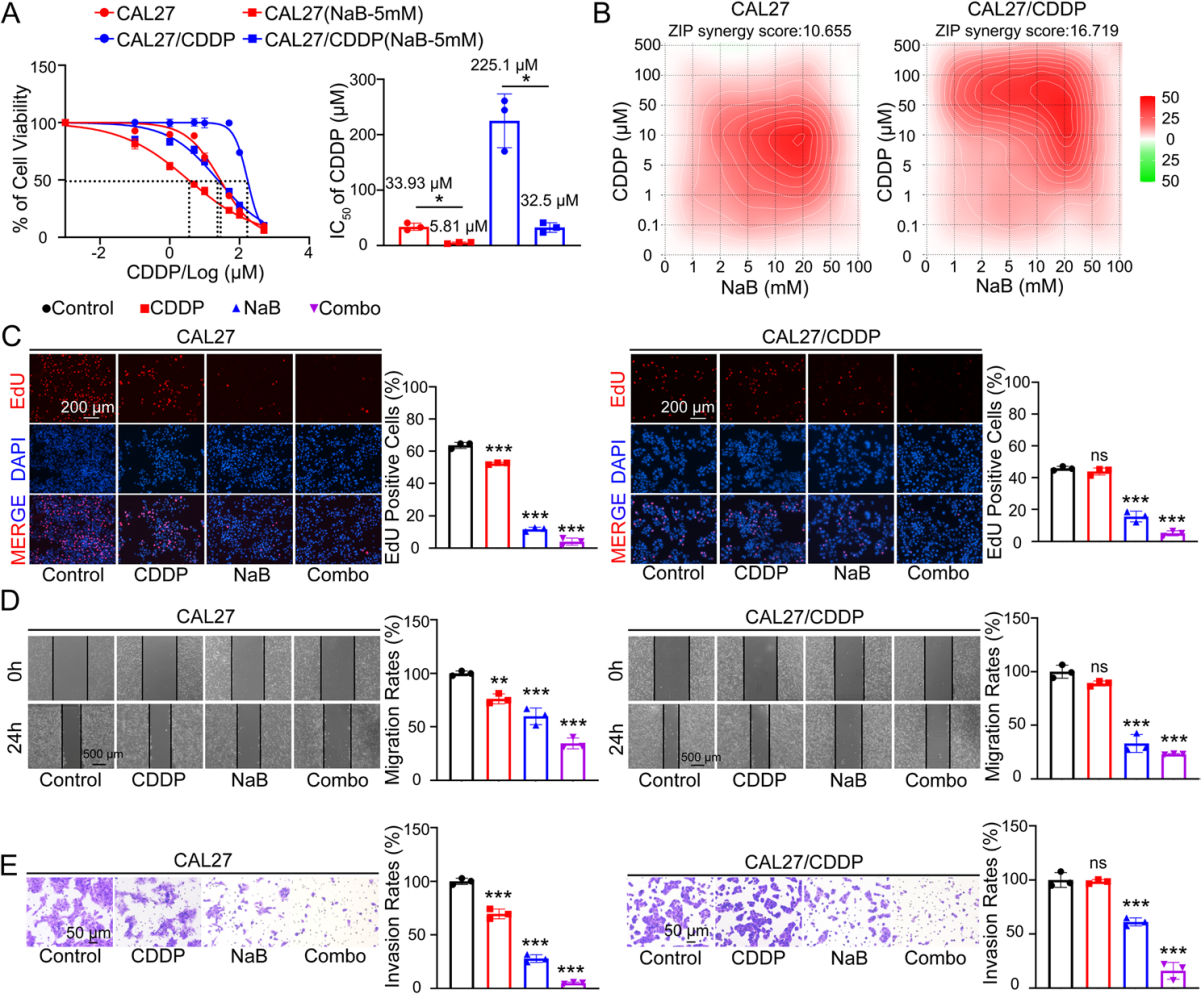
NaB reverses cisplatin resistance of CAL27/CDDP, inhibits CAL27/CDDP and CAL27 cell proliferation, migration, and invasion. **A** The IC_50_ of cisplatin (CDDP) was detected by CCK8 assay in CAL27 and CAL27/CDDP cells with and without 5 mM NaB treatment for 24 h. *, *P* < 0.05. **B** The synergy heatmap of the combination of CDDP and NaB in CAL27 and CAL27/CDDP, calculated using the ZIP model through the SynergyFinder website. (https://synergyfinder.aittokallio.group). **C** Representative images and quantification of EdU positive cell from CAL27 (left panel) and CAL27/CDDP (right panel) after exposure to 5 µM CDDP, 5 mM NaB and their combination for 24 h. Red, EdU. Blue, DAPI. Scale bar, 200 μm. ***, *P* < 0.001. **D** Cell migration characterized through scratch test. Representative scratch images and quantitative analysis in CAL27 (left panel) and CAL27/CDDP (right panel) treated with 5 µM CDDP, 5 mM NaB and their combination for 24 h. Scale bar, 500 μm. ***, *P* < 0.001. **E** Cell invasion characterized through transwell assays. Representative crystal violet staining cell images and quantitative analysis in CAL27 (left panel) and CAL27/CDDP (right panel) treated with 5 µM CDDP, 5 mM NaB and their combination for 24 h. Scale bar, 50 μm. ***, *P* < 0.001

NaB is a well-established histone deacetylase (HDAC) inhibitor that has demonstrated anticancer effects in a variety of malignancies [27–29]. Therefore, we hypothesized that NaB could potentially reverse cisplatin resistance in the OSCC. Different NaB concentrations (0, 1, 2, 5, 10, 50 and 100 mM) were used to treat OSCC cell lines CAL27, CAL27/CDDP, CAL33, HSC3, and SCC15 for 24 h to investigate NaB effect. The CCK8 assay showed that NaB could inhibited cell viability in a dose dependent manner, and 5 mM was the minimum concentration to inhibit cell viability for CAL27, CAL27/CDDP, CAL33, HSC3, and SCC15 (Fig. S1 A). Considering the role of NaB in enhancing chemotherapeutic drugs sensitivity in other tumors [30], we aimed to investigate its role in OSCC. Therefore, these OSCC cell lines CAL27, CAL27/CDDP, CAL33, HSC3, and SCC15 were treated with cisplatin at the gradient concentrations described above, and cisplatin combined with NaB (5 mM), respectively. The cell proliferation of each group was detected 24 h later using CCK8. The results showed that the cell viability of these cells treated with cisplatin combined with NaB was significantly lower than the groups treated with cisplatin alone (Fig. 2A, Fig. S1 B). We also noted that NaB was able to significantly reduce the cisplatin IC_50_ of CAL27/CDDP to near the level of CAL27 (Fig. 2A). To more clearly define the synergistic interaction between NaB and CDDP, we performed a formal synergy analysis using the well-established ZIP model in the SynergyFinder software platform (https://synergyfinder.aittokallio.group) [31, 32]. The ZIP synergy scores were 10.655 in CAL27, 16.719 in CAL27/CDDP, 10.659 in CAL33, 10.013 in HSC3, and 10.238 in SCC15, quantitatively confirming a strong synergistic effect between NaB and CDDP. A positive score indicates a synergistic interaction, where the combined effect is greater than the expected effect if the two drugs were acting independently. The corresponding heatmap clearly illustrates that most dose combinations are represented by distinct red regions, visually corroborating the quantitative synergy scores and confirming a potent synergistic interaction (Fig. 2B, Fig. S1 C).

Then we divided the two cell lines into the following four groups and carried out experiments: control group, cisplatin (5 µM) alone group, NaB (5 mM) alone group and cisplatin combined with NaB group. An EdU proliferation assay was conducted to certify NaB’s effect on cell proliferation. The results showed that treatment with NaB and cisplatin combined with NaB significantly inhibited the proliferation of both CAL27 and CAL27/CDDP (Fig. 2C). In addition, we performed the wound healing assay and transwell assay respectively to assess migration and invasion. The results showed that compared with cisplatin groups, NaB and cisplatin combined with NaB groups inhibited the migration (Fig. 2D) and invasion (Fig. 2E) of CAL27 and CAL27/CDDP more effectively. These results indicated that NaB could reverse cisplatin resistance of CAL27/CDDP and enhance cisplatin sensitivity of CAL27.

### NaB induces ferroptosis to reverse cisplatin resistance of CAL27/CDDP

**Fig. 3:**
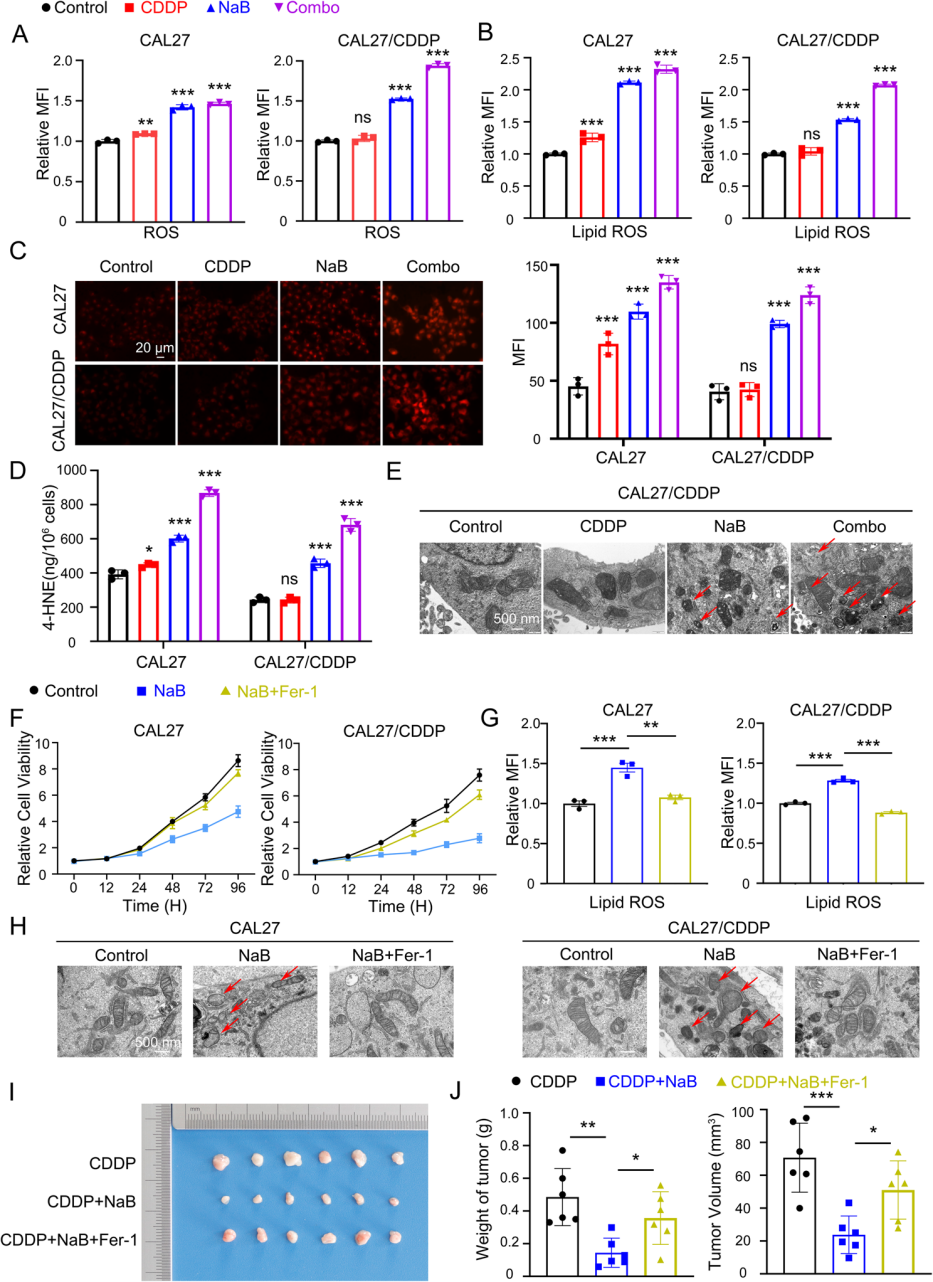
NaB induces ferroptosis to reverse cisplatin resistance of CAL27/CDDP. **A** Quantification of ROS by DHE flow cytometry in CAL27 (left panel) and CAL27/CDDP (right panel) cells. MFI was assessed following 24-hour treatment with 5 µM CDDP, 5 mM NaB and their combination. **, *P* < 0.01 and ***, *P* < 0.001. **B** Quantification of Lipid ROS by C11-BODIPY flow cytometry in CAL27 (left panel) and CAL27/CDDP (right panel) cells. MFI was assessed following 24-hour treatment with 5 µM CDDP, 5 mM NaB and their combination. ***, *P* < 0.001. **C** Representative FerroOrange fluorescent images (left panel) and quantitative analysis of MFI (right panel) in CAL27 and CAL27/CDDP treated with 5 µM CDDP, 5 mM NaB and their combination for 24 h. Scale bar, 20 μm. ***, *P* < 0.001. **D** 4-HNE was quantified using an ELISA assay in CAL27 and CAL27/CDDP cells treated with 5 µM CDDP, 5 mM NaB and their combination for 24 h. *, *P* < 0.05, ***, *P* < 0.001. **E** Representative TEM mitochondria images in CAL27/CDDP cells treated with 5 µM CDDP, 5 mM NaB and their combination for 24 h. Scale bar, 500 nm. **F** The CCK8 assay was used to determine the cell viability of CAL27 (left panel) and CAL27/CDDP (right panel) treated with 5 mM NaB and combination with 10 µM Fer-1 for increased hours. **G** Quantification of Lipid ROS by C11-BODIPY flow cytometry in CAL27 (left panel) and CAL27/CDDP (right panel) cells. MFI was assessed following 24-hour treatment with 5 mM NaB and combination with 10 µM Fer-1. **, *P* < 0.01 and ***, *P* < 0.001. **H** Representative TEM mitochondria images in CAL27 and CAL27/CDDP cells treated with 5 mM NaB and combination with 10 µM Fer-1 for 24 h. Scale bar, 500 nm. **I** The xenograft tumor image of CAL27/CDDP cell line at the endpoint of the in vivo experiment. **J** Tumor weight and volume histogram of CAL27/CDDP xenograft tumors with CDDP (2 mg/kg for once every 3 days, i.p.), NaB (200 mM in drinking water), and Fer-1(5 mg/kg for once every 3 days, i.p.). *, *P* < 0.05, **, *P* < 0.01 and ***, *P* < 0.001

Given the existence of ferroptosis resistance in CAL27/CDDP and the distinct reversal effect of NaB on cisplatin resistance, we sought to further explore lipid peroxidation and ferroptosis level of CAL27/CDDP with or without NaB treatment. Therefore, we detected markers of ferroptosis in both CAL27 and CAL27/CDDP cells by the following assays according to the four groups described above. The results showed that NaB and cisplatin combined with NaB groups significantly increased the generation of ROS compared with CDDP treatment alone in the two cell lines by flow cytometry analysis (Fig. 3A, Fig. S2 A). Similarly, an increase in lipid ROS level was also observed in both the NaB and combination treatment groups through both fluorescence microscope and flow cytometry analysis (Fig. 3B, Fig. S2 B, C). Moreover, we determined intercellular Fe^2+^ concentration in two cell lines. The results implied that, in comparison to CDDP groups, the concentration of Fe^2+^ was significantly higher in both the NaB and cisplatin combined with NaB groups (Fig. 3C). At the same time, ELISA assay also revealed that the lipid peroxidation marker 4-HNE significantly increased in both the NaB and combination treatment groups compared with CDDP treatment alone (Fig. 3D). In addition, we utilized TEM to examine the morphological changes in CAL27/CDDP cells. The cells in NaB and NaB combined with cisplatin groups exhibited shrunken mitochondria with increased membrane density, which is a characteristic morphologic feature of ferroptosis (Fig. 3E).

To confirm that the anti-tumor effects of NaB were primarily mediated through ferroptosis, we treated the cisplatin-sensitive cells CAL27, CAL33, HSC3, SCC15 and cisplatin-resistant cells CAL27/CDDP with NaB in the presence or absence of Fer-1 (10 µM). The CCK8 results demonstrated that Fer-1 significantly rescued cell viability inhibition induced by NaB (Fig. 3F, Fig. S2 D). As measured by C11-BODIPY flow cytometry, the dramatic increase in lipid ROS caused by NaB was effectively suppressed by co-treatment with Fer-1 (Fig. 3G, Fig. S2 E). TEM analysis revealed that the characteristic mitochondrial changes of ferroptosis (e.g., mitochondrial shrinkage, increased membrane density) induced by NaB were largely prevented by Fer-1 (Fig. 3H). Fer-1 almost completely abolished the ability of NaB to inhibit cell migration (Fig. S2 F) and invasion (Fig. S2 G).

To determine whether the in vivo reverse cisplatin resistance effect of NaB is mediated by ferroptosis, we performed a pharmacological rescue experiment using a cisplatin-resistant CAL27/CDDP xenograft model. The animal groups were designed as follows: CDDP alone, CDDP combined with NaB (CDDP + NaB), and CDDP combined with both NaB and Fer-1 (CDDP + NaB+Fer-1) (Fig. 3I-J). Compared with CDDP monotherapy, the combination of CDDP and NaB significantly suppressed tumor growth. Notably, the addition of the ferroptosis inhibitor Fer-1 largely abolished this synergistic effect, restoring tumor size to levels comparable to that of CDDP treatment alone. This conclusion was supported by terminal tumor volume and weight measurements, which were significantly higher in the CDDP + NaB+Fer-1 group than in the CDDP + NaB group. These data provide direct in vivo functional evidence that the chemosensitizing effect of NaB is dependent on the induction of ferroptosis. In conclusion, these results proved that NaB can overcome cisplatin resistance in OSCC by activating ferroptosis.

### The upregulation of POR contributes to NaB-induced ferroptosis in CAL27/CDDP

**Fig. 4:**
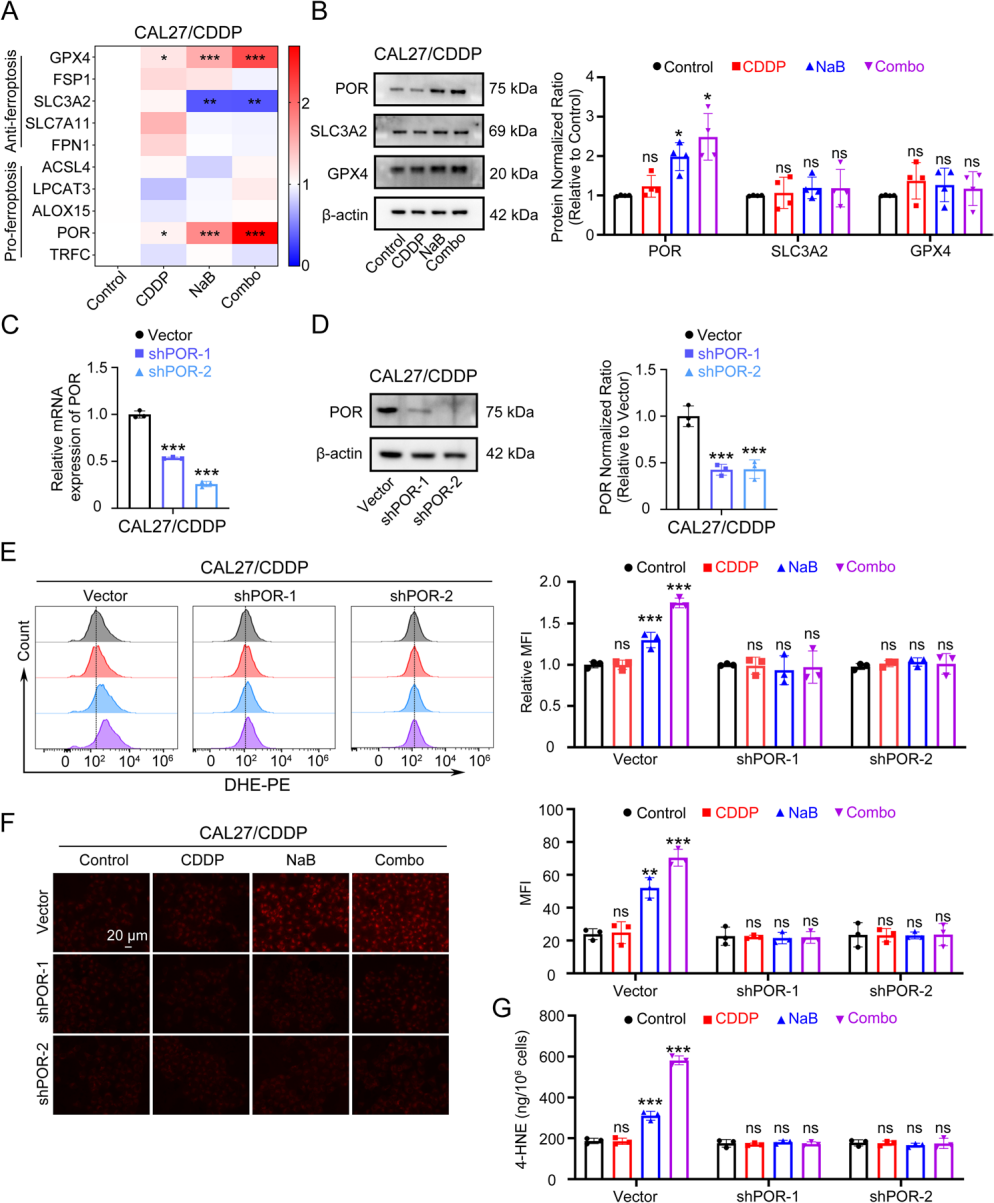
The upregulation of POR contributes to NaB-induced ferroptosis in CAL27/CDDP. **A** Heat maps of mRNA expressions of ferroptosis related genes in CAL27/CDDP treated with 5 µM CDDP, 5 mM NaB and their combination for 24 h. *, *P* < 0.05, **, *P* < 0.01 and ***, *P* < 0.001. **B** Western blot was used to detect the protein expressions of ferroptosis related genes in CAL27/CDDP treated with 5 µM CDDP, 5 mM NaB and their combination for 24 h. The left panel was representative images and the right panel was quantitative analysis. Western blot results were normalized using β-actin as an internal control. *, *P* < 0.05. **C** POR mRNA expression was determined by RT-qPCR to verify the knockdown efficiency in CAL27/CDDP. ***, *P* < 0.001. **D** POR protein expression was determined by western blot to verify the knockdown efficiency in CAL27/CDDP. Western blot results were normalized using β-actin as an internal control. ***, *P* < 0.001. **E** Representative DHE flow cytometry images (left panel) and quantitative analysis of MFI (right panel) in CAL27/CDDP and POR knockdown cell lines, which were treated with 5 µM CDDP, 5 mM NaB and their combination for 24 h. ***, *P* < 0.001. **F** Representative FerroOrange fluorescent images (left panel) and quantitative analysis of MFI (right panel) in CAL27/CDDP and POR knockdown cell lines, which were treated with 5 µM CDDP, 5 mM NaB and their combination for 24 h. Scale bar, 20 μm. **, *P* < 0.01 and ***, *P* < 0.001. **G** 4-HNE was quantified using an ELISA assay in CAL27/CDDP and POR knockdown cell lines, which were treated with 5 µM CDDP, 5 mM NaB and their combination for 24 h. ***, *P* < 0.001

In order to elucidate the molecular mechanism underlying ferroptosis induced by NaB, we assessed the expression of several genes relevant to ferroptosis by using WB and qPCR. Our experimental findings revealed that POR was upregulated at both transcription and translation levels following NaB treatment in both cell lines (Fig. 4A, B, Fig. S3 A, B). While both GPX4 and POR are altered in the CAL27/CDDP cells as shown in the Fig. 1, our key experimental finding was that the therapeutic effect of NaB was specifically mediated through the POR pathway. GPX4 expression did not show a statistically significant change in response to NaB, the upregulation of POR was both pronounced and highly significant at both the mRNA and protein levels. These data strongly suggested that POR, not GPX4, was the primary downstream mediator of NaB’s effect in our model. To investigate the role of POR in NaB-induced ferroptosis, we constructed POR knockdown cell lines through lentivirus infection, and the successful knockdown was confirmed by both mRNA and protein levels (Fig. 4C, D, Fig. S3 C, D). Subsequently, the transfected cells were treated with cisplatin and NaB either alone or in combination according to previously established groups. We then measured the ROS levels, lipid peroxidation metabolites 4-HNE and the intracellular Fe^2+^ concentrations. The results showed that after silencing POR, both NaB and cisplatin combined with NaB treatment failed to elevate ROS (Fig. 4E, Fig. S3 E), Fe^2+^ concentrations (Fig. 4F, Fig. S3 F) and 4-HNE (Fig. 4G, Fig. S3 G) in the two cell lines. These findings strongly supported that the increased expression of POR was crucial for mediating NaB-induced ferroptosis.

### NaB promotes transcription of POR by upregulating EGR1 in CAL27/CDDP

**Fig. 5:**
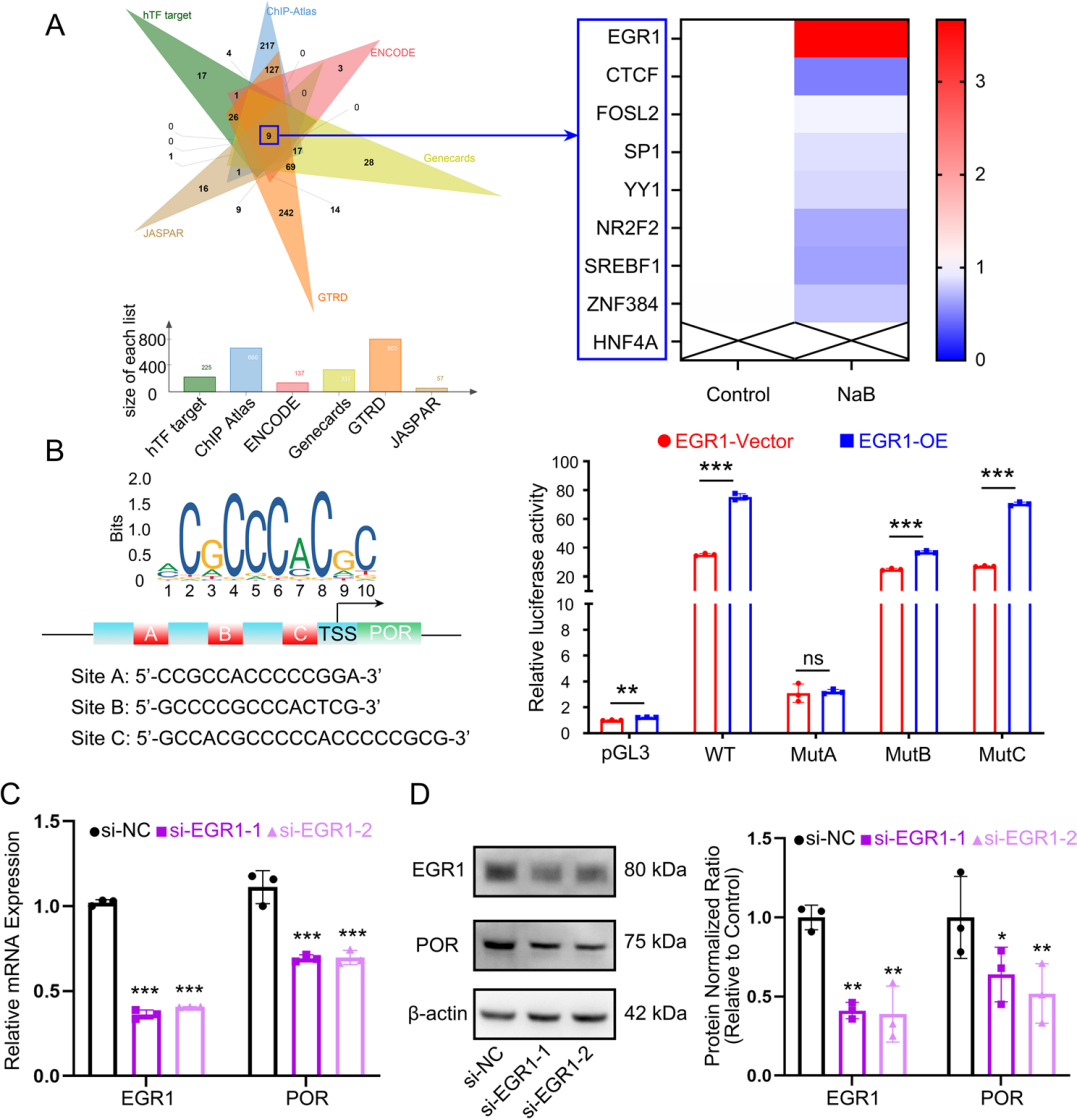
NaB promotes transcription of POR by upregulating EGR1 in CAL27/CDDP. **A** Venn diagram from the six databases identified a total of nine potential transcription factors of POR. **B** The mRNA expression levels of the nine transcription factors following NaB administration in CAl27/CDDP. **C** The schematic diagram showed the locations of the predicted EGR1 binding regions on the POR promoter via the JASPAR website (https://jaspar.elixir.no/). **D** A dual luciferase assay was performed after transfection of the EGR1 overexpression plasmid and the wildtype and mutant sequence of the POR promoter plasmid to detect transcriptional regulation of POR in CAl27/CDDP. WT, wildtype. MutA, Site A mutation. MutB, Site B mutation. MutC, Site C mutation. **, *P* < 0.01. and ***, *P* < 0.001. **E** The mRNA expression levels of EGR1 and POR following EGR1 knockdown with siRNAs in CAl27/CDDP. ***, *P* < 0.001. **F** Effect of EGR1 knockdown on EGR1 and POR protein levels in CAl27/CDDP was determined by western blot. Western blot results were normalized using β-actin as an internal control. *, *P* < 0.05 and **, *P* < 0.01

To define the mechanisms by which NaB upregulates the expression of POR, we integrated hTFtarget (https://guolab.wchscu.cn/hTFtarget/), ChIP Atlas (https://chip-atlas.org/), ENCODE (https://www.encodeproject.org/), GeneCards (https://www.genecards.org/), GTRD (https://gtrd.biouml.org/), UCSC JASPAR (https://jaspar.elixir.no/) databases to predict potential transcription factors associated with POR. The upset plot of the Venn diagram encompassing the six databases identified a total of nine candidate transcription factors, including EGR1, CTCF, FOSL2, SP1, YY1, NR2F2, SREBF1, ZNF384, HNF4A (Fig. 5A).

Subsequently, we compared the mRNA expression levels of the nine transcription factors following NaB administration in CAl27/CDDP cells. The results indicated that mRNA expression of EGR1 was significantly elevated, while HNF4A showed no expression, and other transcription factors exhibited either unchanged or decreased mRNA expression after the treatment of NaB (Fig. 5B). The consistent increase in EGR1 alongside POR suggests that EGR1 may function as a transcription factor for POR. To validate this hypothesis, we obtained 3 high-confidence sequences from the promoter region of POR that are likely to bind with EGR1 via the JASPAR website (https://jaspar.elixir.no/) (Fig. 5C). To determine which predicted EGR1 binding site is functional, we performed a dual-luciferase reporter assay. As shown in Fig. 5D, overexpression of EGR1 significantly enhanced the transcriptional activity of the reporter plasmid containing the wild-type POR promoter sequence. Importantly, when the core sequence of Site A (5’-CCGCCACCCCCGGA-3’) was mutated (to 5’-AATAACAAAAATTC-3’), this activation was completely abolished. In contrast, mutations at site B and C had no significant effect on EGR1-induced promoter activity. These results demonstrated that Site A was both necessary and sufficient for the transactivation of the POR promoter by EGR1 (Table S4). These findings collectively support our conclusion that EGR1 facilitates the transcriptional activation of POR, and their binding site corresponds to the sequence at Site A.

To further validate the role of EGR1 in NaB-mediated regulation of POR expression, we employed small interfering RNA (siRNA) technology to specifically knockdown EGR1 and examined its effect on POR expression levels. CAL27/CDDP cells were transfected with either EGR1-targeting siRNA or negative control siRNA (si-NC), followed by qPCR and Western blot analyses to assess changes in EGR1 and POR expression at both mRNA and protein levels. As expected, EGR1 was successfully knockdown at both transcription and translation levels (*P* < 0.01, Fig. 5E, F). Further analysis revealed that the reduction of EGR1 in CAL27/CDDP cells led to significant decreases in POR expression at both mRNA and protein levels (*P* < 0.05, Fig. 5E, F). These consistent findings strongly support EGR1’s role as a transcriptional activator of POR.

### NaB upregulates EGR1 via HDAC9 inhibition-mediated H3K27 acetylation

**Fig. 6:**
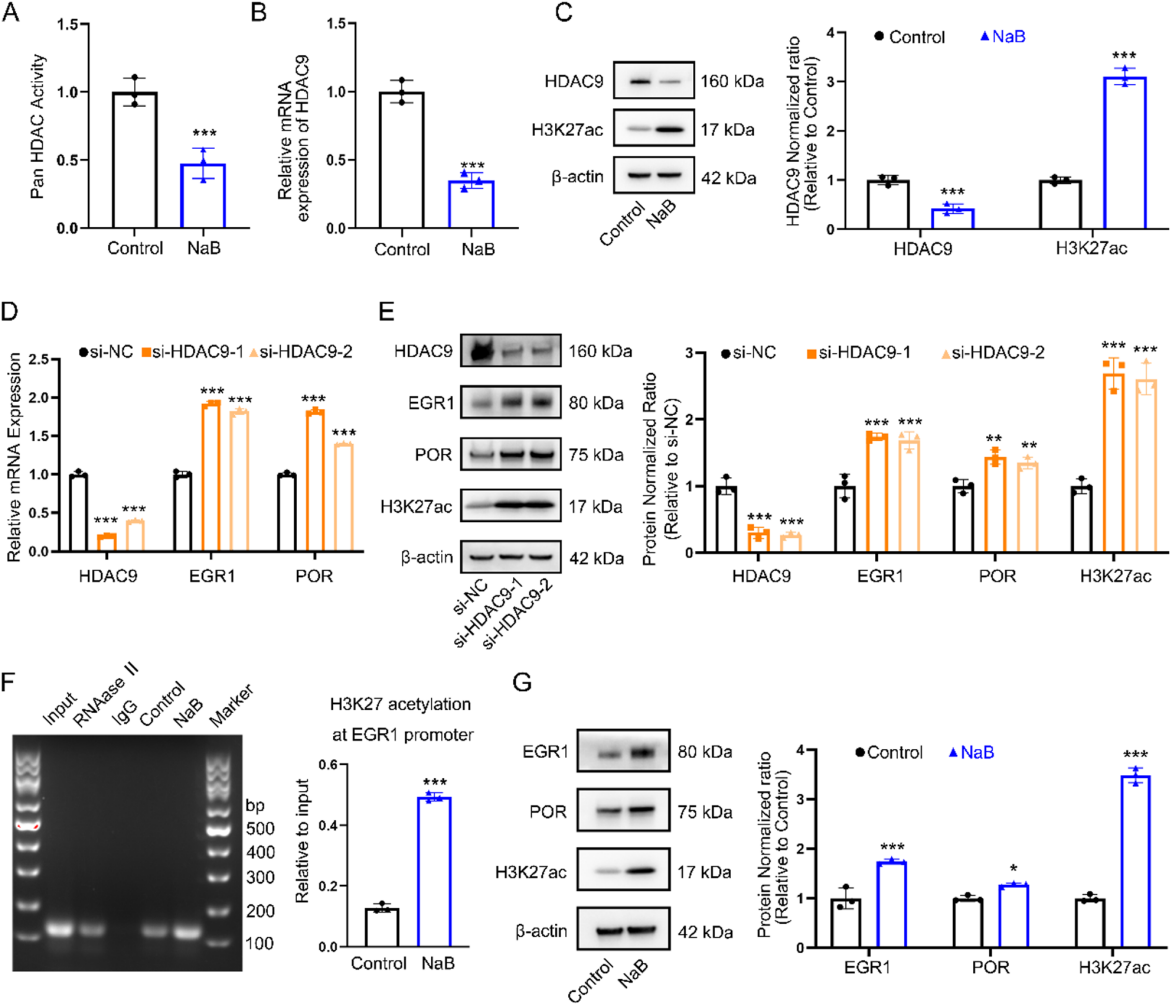
NaB upregulates EGR1 via HDAC9 inhibition-mediated H3K27 acetylation. **A** Effect of NaB on pan-HDAC activity in CAL27/CDDP. ***, *P* < 0.001. **B** The HDAC9 mRNA expression levels following NaB administration in CAl27/CDDP. ***, *P* < 0.001. **C** Effect of NaB on HDAC9 and H3K27ac protein levels in CAl27/CDDP was determined by western blot. Western blot results were normalized using β-actin as an internal control. ***, *P* < 0.001. **D** The mRNA expression levels of HDAC9, EGR1 and POR following HDAC9 knockdown in CAl27/CDDP. ***, *P* < 0.001. **E** Effect of HDAC9 knockdown on HDAC9, EGR1, POR, and H3K27ac protein levels in CAl27/CDDP was determined by western blot. Western blot results were normalized using β-actin as an internal control. **, *P* < 0.01 and ***, *P* < 0.001. **F** Agarose gel electrophoresis of sonicated chromatin of CAl27/CDDP treated with NaB for 24 h (left panel). H3K27 acetylation levels at EGR1 promoter of CAL27/CDDP treated with NaB by ChIP-qPCR (right panel). β-actin promoter was used as an internal control. ***, *P* < 0.001. **G** Effect of NaB on EGR1, POR and H3K27ac protein levels in CAl27/CDDP was determined by western blot. Western blot results were normalized using β-actin as an internal control. *, *P* < 0.05 and ***, *P* < 0.001

Given that NaB can modulate signal transduction by inhibiting histone deacetylases (HDACs), we further investigated whether it might upregulate POR by blocking HDACs. Pan-HDAC activity assays revealed that NaB downregulated overall HDAC activity (Fig. 6A). To directly rule out the contribution of the major known HDAC-independent targets of butyrate, namely the G protein-coupled receptors (GPCRs) GPR41, GPR43, and GPR109a [28], we treated CAL27/CDDP cells with specific inhibitors of GPR41 (β-hydroxybutyric acid), GPR43 (GLPG0974), and GPR109a (mepenzolate bromide) in combination with NaB administration. Our results clearly demonstrated that blocking these GPCR signaling pathways did not attenuate NaB-induced ferroptosis (Fig. S4 A). Following NaB treatment in CAL27/CDDP cells, we observed a significant reduction in both mRNA and protein levels specifically for HDAC9 among all subtypes of HDACs (Fig. 6B, C). Alterations in the mRNA levels of the remaining HDACs were shown in the supplementary figures (Fig. S4 B). Interestingly, we also found that the application of NaB could significantly promote H3K27 acetylation (Fig. 6C).

To investigate the function of HDAC9, we silenced HDAC9 in CAL27/CDDP cells using siRNA technology. qPCR and Western blot analysis confirmed efficient HDAC9 knockdown (*P* < 0.001, Fig. 6D, E). Notably, HDAC9 knockdown significantly increased both POR and EGR1 expression at both the mRNA and protein levels (*P* < 0. 01, Fig. 6D, E), demonstrating that HDAC9 normally functions to suppress the transcriptional activity of these genes. Crucially, in CAL27/CDDP cells in which HDAC9 was silenced, subsequent application of NaB did not produce a further significant increase in the mRNA levels of EGR1 or POR (Fig. S4 D). Furthermore, we observed a marked increase in H3K27 acetylation following HDAC9 knockdown (Fig. 6E), suggesting that HDAC9 may target H3K27 deacetylation to exert transcriptional repression.

Subsequently, we conducted chromatin immunoprecipitation (ChIP) qPCR to assess H3K27ac enrichment at the EGR1 promoter after NaB treatment in CAL27/CDDP cells. The results of ChIP assay showed that NaB significantly increased H3K27ac at EGR1 promoter (Fig. 6F) and elevated EGR1 protein expression, which was positively correlated with POR (Fig. 6G). These findings collectively establish HDAC9 as a key epigenetic regulator that controls POR and EGR1 expression via modification of H3K27 acetylation status in CAL27/CDDP cells. Mechanically, these findings suggest that NaB inhibits HDAC9 to enrich H3K27ac at the EGR1 promoter, which elevate EGR1 expression, subsequently upregulating POR and promoting ferroptosis in in CAL27/CDDP cells.

### NaB combined with CDDP effectively suppresses the proliferation of CDDP resistant tumors in vivo

**Fig. 7:**
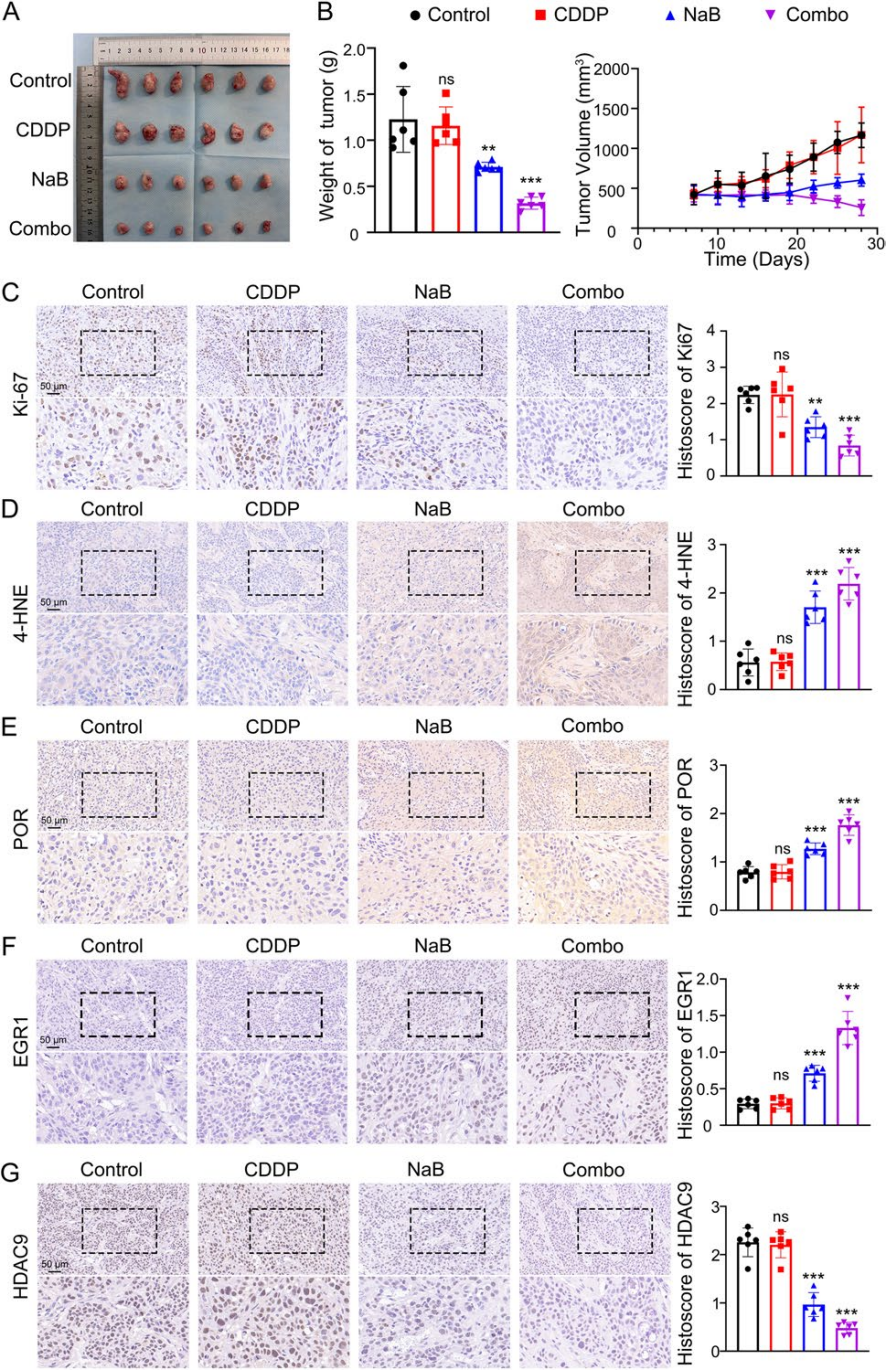
NaB combined with CDDP effectively suppresses the proliferation of CDDP resistant tumors in vivo. **A** The xenograft tumor image of CAL27/CDDP cell line at the endpoint of the in vivo experiment. **B** Tumor weight histogram (left panel) and tumor volume line chart (right panel) of CAL27/CDDP xenograft tumors treated with CDDP (2 mg/kg for once every 3 days, i.p.), NaB (200 mM in drinking water) and their combined treatment. **, *P* < 0.01 and ***, *P* < 0.001. Representative IHC images of Ki-67 (**C**), 4-HNE (**D**), POR (**E**), EGR1 (**F**) and HDAC9 (**G**) and quantitative analysis in CAL27/CDDP xenograft tumors treated with CDDP, NaB and their combined treatment. Scale bar, 50 μm. **, *P* < 0.01 and ***, *P* < 0.001

The xenograft models bearing CAL27/CDDP cells were employed to further investigate whether the inhibition of tumor growth by NaB in vivo was associated with ferroptosis. According to tumor images and tumor weight analysis, NaB restrained cisplatin resistant tumor growth and cisplatin also showed significant inhibitory effect on cisplatin resistant tumor when combined with NaB. (Fig. 7A, B). A reduction in tumor volume indicated that tumor proliferation was significantly inhibited after NaB treatment and cisplatin combined with NaB treatment (Fig. 7B). Ki-67, a nuclear protein linked to cell cycle progression, reflects the proportion of cells within the proliferative phase [33]. We noted a significant decrease in the percentage of Ki67-positive cells following NaB intervention, particularly when combined with cisplatin, suggesting that NaB effectively suppressed tumor proliferation in vivo and enhanced the therapeutic effect of cisplatin (Fig. 7C). To further elucidate the relationship between NaB-induced tumor growth inhibition and ferroptosis, immunohistochemistry (IHC) analysis was conducted to assess the expression levels of 4-HNE, POR, EGR1 and HDAC9 in tumor tissues. Notably, we observed a significant increase in 4-HNE expression after NaB treatment especially when used alongside cisplatin indicating that NaB induced ferroptosis in cisplatin resistant tumor tissues (Fig. 7D). Consistent with our in vitro results, these in vivo findings further demonstrated that NaB inhibited tumor proliferation through induction of ferroptosis via upregulation of POR (Fig. 7E) and EGR1 (Fig. 7F) expression, and inhibition of HDAC9 expression (Fig. 7G). Body weight curves of each group demonstrated the excellent biocompatibility of NaB (Fig. S5 A). Serum biochemical indices (ALT, AST, BUN, Cr) of the mice also demonstrated the safety of the strategy (Fig. S5 B). Additionally, heart, liver, spleen, lung and kidney tissues were collected to evaluate the safety profile of NaB. Hematoxylin and eosin (H&E) staining revealed no significant alterations to organ morphology attributable to either drug indicating its safety as a viable intervention strategy. (Fig. S5 C).

## Discussion

Oral squamous cell carcinoma (OSCC) patients in advanced stages are predominantly treated with combination therapy based on cisplatin or fluoropyrimidines [34]. However, chemoresistance remains a significant factor contributing to poor prognosis in advanced and recurrent OSCC patients [35]. Regrettably, the underlying mechanisms responsible for cisplatin resistance have yet to be thoroughly investigated. In this study, we elucidate the effects of combined treatment with sodium butyrate (NaB) and cisplatin in one cell model of OSCC. Our findings demonstrate that the application of NaB effectively inhibited cell proliferation and tumor growth in xenograft models. Furthermore, NaB induced ferroptosis in a cisplatin-resistant OSCC cell line both *in vitro and in vivo*. Mechanistically, we discovered that NaB upregulates POR expression through an HDAC9/EGR1 mediated translational increase to participate in lipid peroxidation. Taken together, here we report that the novel HDAC9/EGR1/POR signaling pathway involve in the combination of NaB and cisplatin treatment effectively reverses chemoresistance in the OSCC model CAL27/CDDP by inducing ferroptosis. Our findings further strengthened the notion that NaB could serve as a promising pharmacological strategy for various types of chemoresistant cancers.

Ferroptosis is a novel form of programmed cell death charactered by iron-dependent lipid peroxidation of the cellular membrane [36]. Currently, numerous studies on ferroptosis in cancer support the innovative concept that inducing ferroptosis may eventually aid in eradicating malignant tumors resistant to chemotherapy [37]. For instance, the combination of shikonin and cisplatin has been shown to overcome cisplatin resistance by promoting Fe^2+^ accumulation and subsequently inducing ferroptosis in ovarian cancer [38]. Similarly, previous research indicated that genes associated with negative regulation of ferroptosis are upregulated in chemotherapy-resistant gastric cancer cells [39]. In this study, we compared the level of ferroptosis markers between the cisplatin-resistant OSCC cells and their parental cells. The cisplatin-resistant cells exhibited reduced lipid peroxidation (lipid ROS), reactive oxygen species (ROS) and Fe^2+^ concentrations relative to the parental cells, implying diminished ferroptosis. Moreover, administration of a ferroptosis inhibitor (Fer-1) markedly potentiated cellular susceptibility to cisplatin, as evidenced by a significant decrease in half-maximal inhibitory concentration (IC₅₀). Consistently, co-treatment with a specific ferroptosis inducer (erastin) substantially attenuated cisplatin cytotoxicity and restored cell viability. The results demonstrate that pharmacologic modulation of ferroptosis directly governs chemosensitivity to cisplatin in OSCC models. Therefore, these findings suggest that the induction of ferroptosis may represent a novel approach for enhancing the sensitivity of chemotherapy and reversing chemoresistance in OSCC.

Recently, emerging evidence has revealed that sodium butyrate (NaB), a type of short-chain fatty acid (SCFA), plays an anti-tumor role across various cancer types. For instance, it has been reported that sodium butyrate can enhance the anti-tumor activity of cytotoxic T lymphocytes (CTLs) and anti-tumor chimeric antigen receptor (CAR) T cells in melanoma and pancreatic cancer [40]. In hepatic cancer, NaB promotes ROS release via miR-22/SIRT-1 axis to induce apoptosis, thereby inhibiting tumor progression [41]. Additionally, NaB restrains the growth of colorectal cancer by disrupting the aerobic glycolysis mediated by SIRT4/HIF-1α [42]. Consequently, the precise role of NaB in cancer therapy is complex and varies depending on factors such as cancer type, treatment strategy and other intricate biological elements. Although it has been demonstrated that NaB exerts anti-tumor effects both in vitro and in vivo [43], it remains unclear the effect of NaB on chemoresistant tumor. As ferroptosis is a regulated cell death process known to be influenced by epigenetic mechanisms [44–48], we sought to investigate whether NaB could modulate ferroptosis sensitivity in our CDDP-resistant OSCC model. In this study, we present finding regarding the impact of NaB on ferroptosis in OSCC and identify NaB as an effective anti-chemoresistant SCFA. Our research confirms a significant observation that NaB not only inhibits proliferation, migration and invasion, but also induces ferroptosis in both cisplatin-resistant cells and parental cells. Notably, compared to treatment with cisplatin alone, the addition of NaB effectively reverses resistance to cisplatin in cisplatin-resistant cells while significantly enhancing the sensitivity to cisplatin in parental cells, which suggests that NaB is able to sensitize against both intrinsic and acquired resistance to CDDP. Consistent with our results are several lines of evidence suggesting that NaB can increase tumor sensitivity to chemotherapy drugs. In colorectal cancer, NaB promotes the cancer cell death while improving the therapeutic sensitivity to 5-fluorouracil (5-FU) [49]. Furthermore, NaB facilitates the efficacy of oxaliplatin chemotherapy by directly promoting CD8^+^ T cell immune responses through an ID2-dependent mechanism [30]. Similar to our findings, another study indicates that NaB stimulates ferroptosis and inhibits tumor progression in endometrial cancer [50]. Together with previous studies, our findings clearly suggest the potential therapeutic application of NaB for treating OSCC with anti-chemoresistance.

Lipid peroxides build up and redox balance is disturbed in ferroptosis. Cytochrome P450 oxidoreductase (POR), an NADPH-dependent oxidoreductase containing flavin mononucleotide, plays a crucial role in promoting ferroptosis. Unlike acyl-coenzyme A synthetase long-chain family member 4 (ACSL4) and lysophosphatidylcholine acyltransferase (LPCAT3), which reshape the polyunsaturated lipidome, POR is directly involved in lipid peroxidation by transferring electrons to downstream effectors, thereby accelerating the cycle between Fe(ii) and Fe(iii) and inducing ferroptosis [16]. In our study, NaB significantly increased the expression of POR at both mRNA and protein levels, suggesting that NaB effectively promoted lipid peroxidation in the OSCC CAL27 cells. When cisplatin-resistant cells were co-treated with NaB and cisplatin, POR expression was markedly up-regulated compared to treatment with either NaB or cisplatin alone. These findings may provide a foundation for the application of NaB in combination with cisplatin for treating OSCC patients. Furthermore, the effect of NaB in promoting ferroptosis on OSCC cells was reversed upon depletion of POR, underscoring its critical role in mediating NaB-induced ferroptosis.

The effects of POR vary across different tumors. For instance, high expression levels of POR are significantly associated with improved overall survival (OS) in patients with hepatitis B virus (HBV)-related hepatocellular carcinoma (HCC), indicating that POR may serve as a potential prognostic biomarker following hepatectomy [51]. In head and neck squamous cell carcinoma (HNSCC), POR was identified as a potential predictive biomarker of hypoxia-activated prodrugs (HAP) sensitivity, which could be used to evaluate the anti-tumor efficacy of HAP [52]. Consistent with our findings, hepatic nuclear factor 4 alpha (HNF4A) has been shown to promote the expression POR and subsequently induce ferroptosis in lung adenocarcinoma [53]. However, the precise role of POR in OSCC remains inadequately explored. In this study, we observed that ferroptosis and POR expression were elevated in cisplatin-resistant cells as well as parent cells after NaB treatment. Mechanistically, we confirmed for the first time that EGR1 acts as a positive transcription factor of POR and promotes the expression of POR. NaB functions as a histone deacetylase (HDAC) inhibitor [54], directly inhibiting HDAC9 and resulting in the enrichment of H3K27ac at the promoter of EGR1. This process leads to an increased expression of EGR1, which in turn promotes the expression of POR and subsequently induces ferroptosis. These findings suggest that targeting POR to modulate ferroptosis levels may represent a promising therapeutic strategy for overcoming chemotherapy resistance. Future studies involving well-designed prospective cohorts with detailed chemotherapy response records are warranted to clinically validate HDAC9, EGR1, and POR as robust prognostic and predictive biomarkers for OSCC.

From a therapeutic perspective, compared to previously identified ferroptosis activators or HDAC inhibitors, NaB is a more attractive alternative because it is a naturally occurring, inexpensive and safe metabolite. However, future research should focus on investigating whether the levels of butyrate in the saliva of OSCC patients exhibiting chemotherapy resistance are altered, and whether salivary butyrate concentrations could serve as potential biomarkers for predicting the therapeutic outcomes of chemotherapy agents. Finally, we acknowledge a limitation of this study. Our conclusions are primarily derived from a single isogenic cell line pair (CAL27 and its CDDP-resistant derivative). While this model provides a controlled system for mechanistic dissection, the generalizability of the HDAC9-EGR1-POR pathway requires further validation in future studies. These should include investigations in other OSCC cell lines with innate or acquired cisplatin resistance, as well as more complex models such as patient-derived organoids (PDOs) or xenografts (PDXs), to confirm its broader clinical relevance.

## Conclusion

**Fig. 8:**
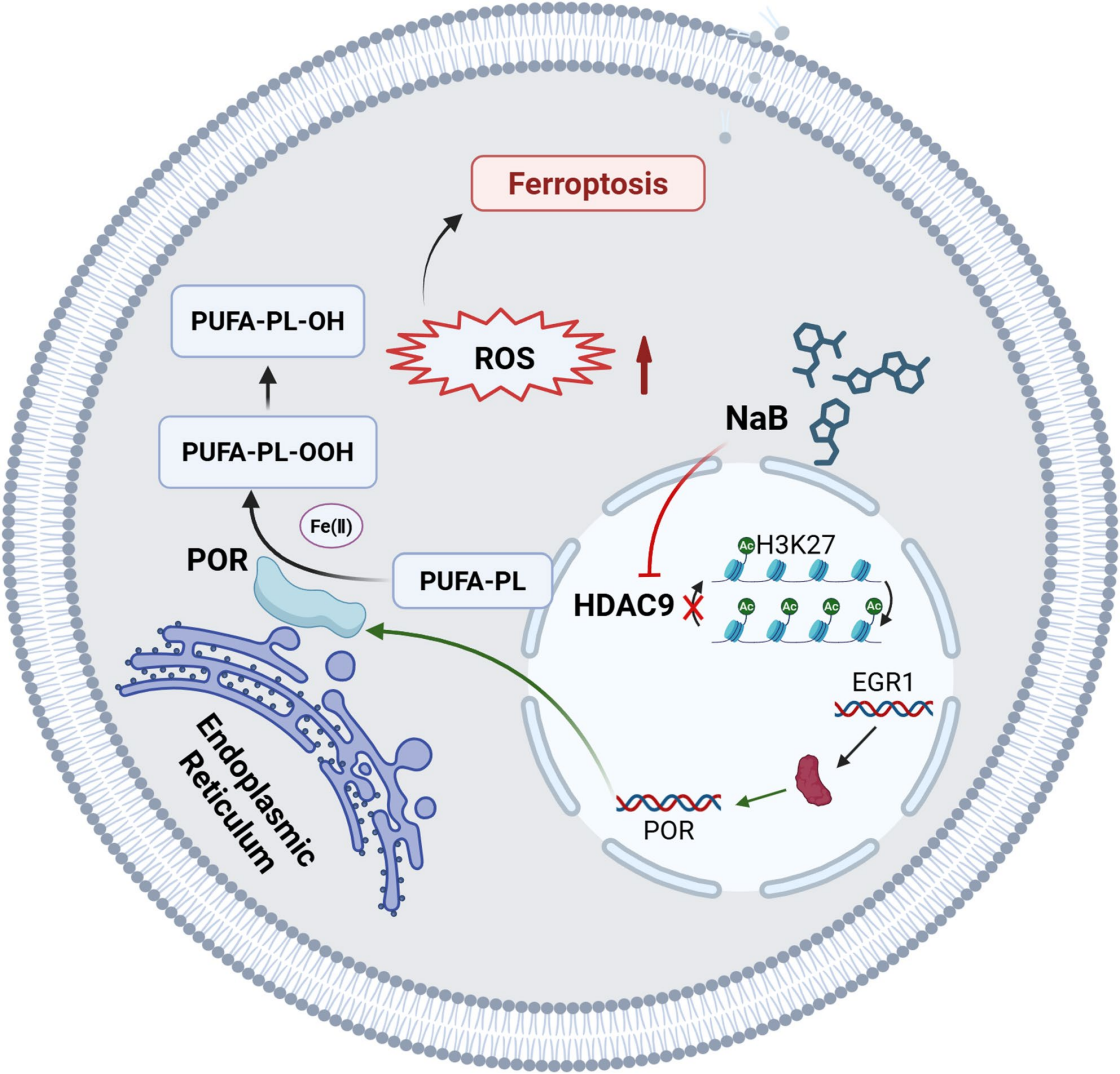
Model diagram of the role of HDAC9/EGR1/POR signal pathway in NaB-mediated ferroptosis in cisplatin- resistant oral squamous cell carcinoma

Collectively, we found that inactivated ferroptosis plays an important role in the resistance of the OSCC CAL27 cell line to cisplatin. Especially, the current study highlighted a novel finding: the application of NaB sensitizes cisplatin-resistant OSCC cells to cisplatin by promoting ferroptosis through HDAC9/EGR1/POR signaling pathway (Fig. 8). Consequently, our data may provide a promising therapeutic potential for reversing cisplatin resistance in the treatment of OSCC patients.

In cisplatin-resistant OSCC, sodium butyrate (NaB) inhibits HDAC9 activity, resulting in increased H3K27 acetylation enrichment at the EGR1 promoter region and subsequent upregulation of EGR1 expression. As a transcription factor, EGR1 enhances POR expression. POR promotes iron redox cycling between Fe(II) and Fe(III) within cytochrome P450 (CYP) enzymes, initiating and propagating lethal lipid peroxidation. This process generates reactive lipid radicals that disrupt cellular membranes, thereby inducing ferroptosis and ultimately reversing cisplatin resistance (created at https://BioRender.com).

## Supplementary Information

Supplementary Material 1.

Supplementary Material 2.

Supplementary Material 3.

Supplementary Material 4.

Supplementary Material 5.

## Data Availability

The datasets used and/or analyzed are available from the corresponding author on reasonable request.

## Abbreviations

OSCC: Oral squamous cell carcinoma
NaB: Sodium butyrate
HDAC: Histone deacetylase
CDDP: Cisplatin
POR: Cytochrome P450 oxidoreductase
EGR1: Early growth response protein 1
FBS: Fetal bovine serum
DHE: Dihydroethidium
ROS: Reactive oxygen species
TEM: Transmission eletron microscopy
Qpcr: Quantitative real-time PCR
ChIP: Chromatin immunoprecipitation
HE: Histological hematoxylin-eosin
IHC: Immunohistochemistry
SCFA: Short chain fatty acids
lipid ROS: Lipid peroxides
4-HNE: 4-hydroxy-2-nonenal
GPX4: Glutathione peroxidase 4
