# Supplementary material for: Uncovering a novel treatment strategy: sodium butyrate overcomes cisplatin resistance in the oral squamous cell carcinoma by inducing ferroptosis
Source: J Exp Clin Cancer Res. 2026 Feb 16;45:66. doi: 10.1186/s13046-026-03663-0 (PMC12980862; doi:10.1186/s13046-026-03663-0)
Supplement: Supplementary file 1 — Supplementary Material 1. [file 13046_2026_3663_MOESM1_ESM.docx]

**Supplementary Figure Legend**

**
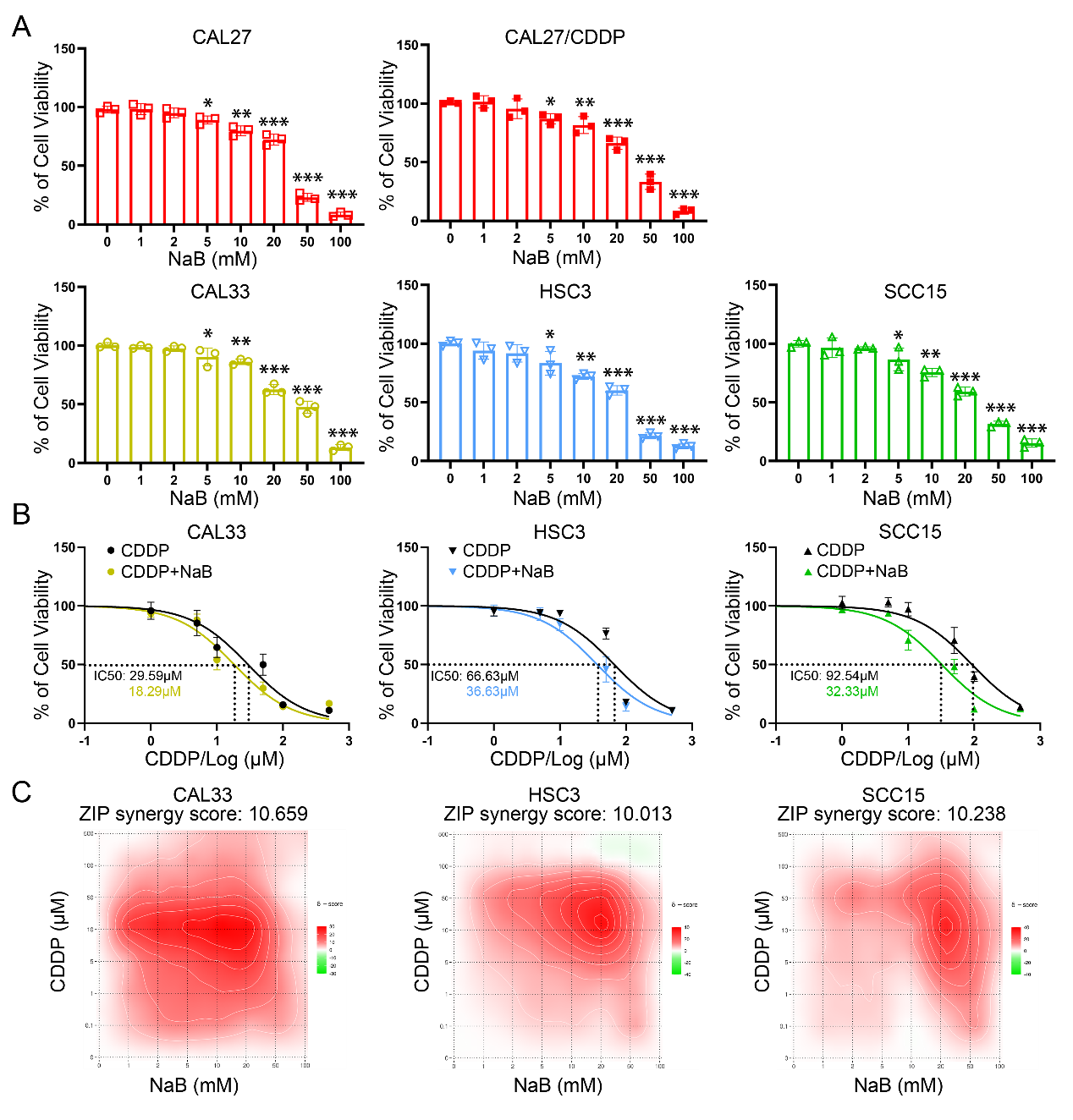
**

**Figure S1. Effect of NaB on cell viability assessed by CCK-8 assay.**

**(A)** The CCK8 assay was used to determine the cell viability of CAL27, CAL27/CDDP, CAL33, HSC3 and SCC15 cells treated with different NaB doses (0, 1, 2, 5, 10, 15,20 and 100 mM) in 24 hours. *, *P*<0.05, **, *P*<0.01 and ***, *P*<0.001.

**(B)** The IC_50_ of cisplatin (CDDP) was detected by CCK8 assay in CAL33, HSC3 and SCC15 cells with and without 5mM NaB treatment for 24 hours.

**(C)** The synergy heatmap of the combination of CDDP and NaB in CAL33, HSC3 and SCC15 cells, calculated using the ZIP model through the SynergyFinder website. (https://synergyfinder.aittokallio.group)

**
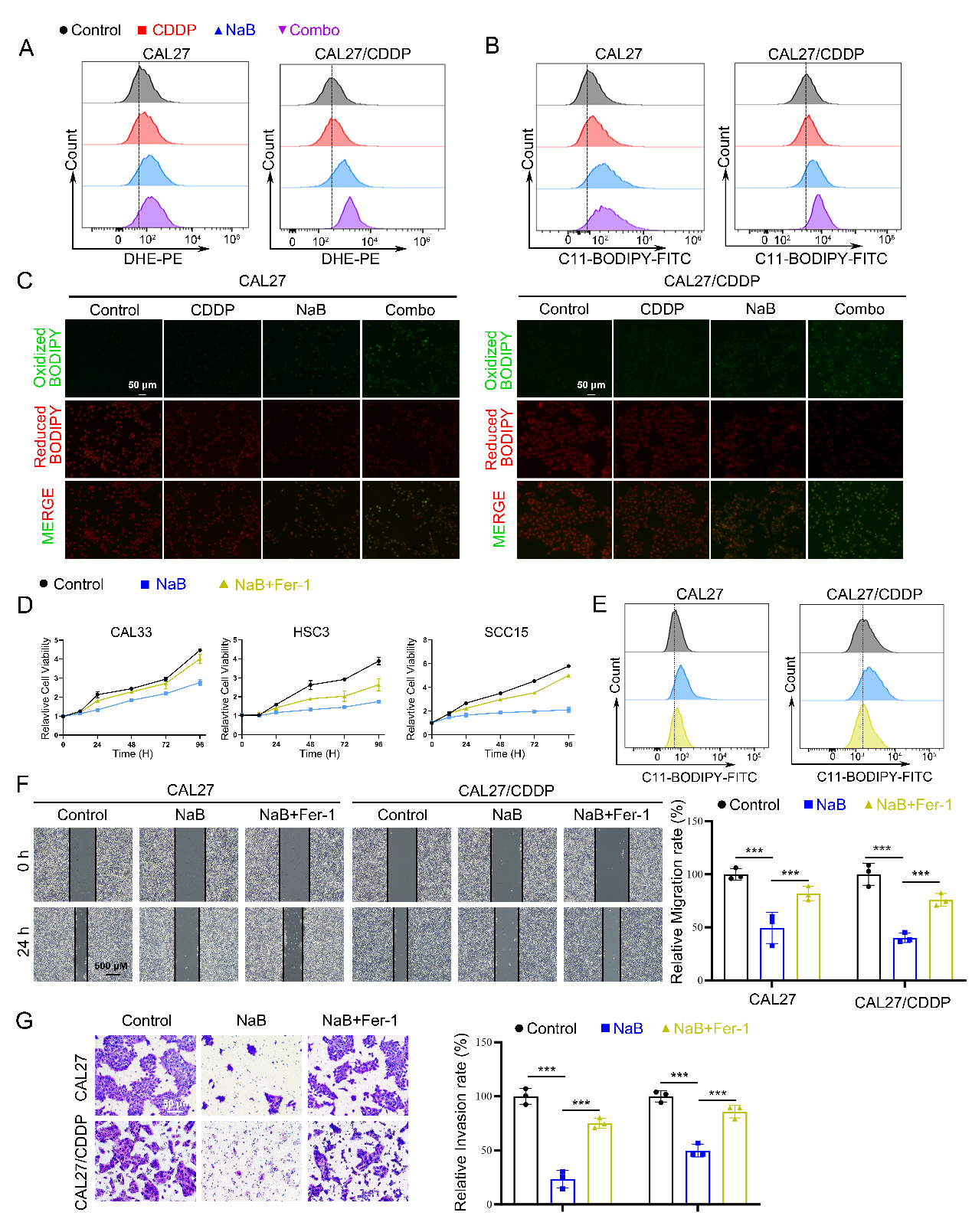
**

**Figure S2. NaB induces ferroptosis to reverse cisplatin resistance of CAL27/CDDP.**

**(A)** Representative DHE flow cytometry images in CAL27 (left panel) and CAL27/CDDP (right panel) treated with 5 μM CDDP, 5 mM NaB and their combination for 24 hours.

**(B)** Representative C11-BODIPY flow cytometry images in CAL27 (left panel) and CAL27/CDDP (right panel) treated with 5 μM CDDP, 5 mM NaB and their combination for 24 hours.

**(C)** Representative C11-BODIPY fluorescent images in CAL27 (left panel) and CAL27/CDDP (right panel) treated with 5 μM CDDP, 5 mM NaB and their combination for 24 hours.

**(D)** The CCK8 assay was used to determine the cell viability of CAL33 (left panel), HSC3 (middle panel) and SCC15 (right panel) treated with 5 mM NaB and combination with 10 μM Fer-1 for increased hours.

**(E)** Representative C11-BODIPY flow cytometry images in CAL27 (left panel) and CAL27/CDDP (right panel) treated with 5 mM NaB and combination with 10 μM Fer-1 for 24 hours.

**(F)** Cell migration characterized through wound healing assays. Representative images and quantitative analysis in CAL27 and CAL27/CDDP treated with 5 mM NaB and 10 μM Fer-1 for 24 hours. Scale bar, 500 μm. ***, *P*<0.001.

**(G)** Cell invasion characterized through transwell assays. Representative crystal violet staining cell images and quantitative analysis in CAL27 and CAL27/CDDP treated with 5 mM NaB and 10 μM Fer-1 for 24 hours. Scale bar, 50 μm. ***, *P*<0.001.


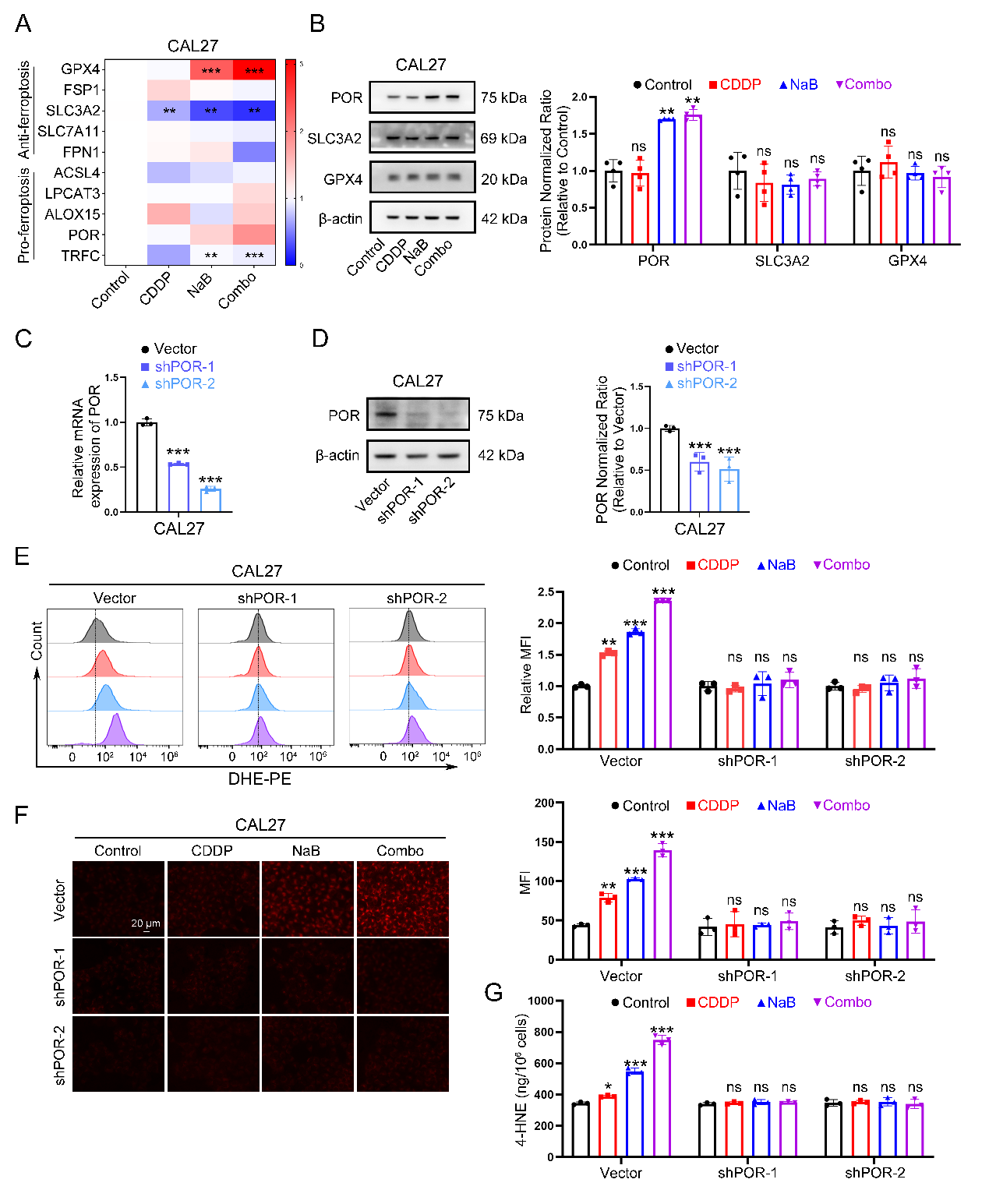


**Figure S3. The upregulation of POR contributes to NaB-induced ferroptosis in CAL27.**

**(A)** Heat maps of mRNA expressions of ferroptosis related genes in CAL27 treated with 5 μM CDDP, 5 mM NaB and their combination for 24 hours. **, *P*<0.01 and ***, *P*<0.001.

**(B)** Western blot was used to detect the protein expressions of ferroptosis related genes in CAL27 treated with 5 μM CDDP, 5 mM NaB and their combination for 24 hours. The left panel was representative images and the right panel was quantitative analysis. Western blot results were normalized using β-actin as an internal control. **, *P*<0.01.

**(C)** POR mRNA expression was determined by RT-qPCR to verify the knockdown efficiency in CAL27. ***, *P*<0.001.

**(D)** POR protein expression was determined by western blot to verify the knockdown efficiency in CAL27. Western blot results were normalized using β-actin as an internal control. ***, *P*<0.001.

**(E)** Representative DHE flow cytometry images (left panel) and quantitative analysis of MFI (right panel) in CAL27 and POR knockdown cell lines, which were treated with 5 μM CDDP, 5 mM NaB and their combination for 24 hours. ***, *P*<0.001.

**(F)** Representative FerroOrange fluorescent images (left panel) and quantitative analysis of MFI (right panel) in CAL27 and POR knockdown cell lines, which were treated with 5 μM CDDP, 5 mM NaB and their combination for 24 hours. Scale bar, 20 μm. **, *P*<0.01 and ***, *P*<0.001.

**(G)** 4-HNE was quantified using an ELISA assay in CAL27 and POR knockdown cell lines, which were treated with 5 μM CDDP, 5 mM NaB and their combination for 24 hours. *, *P*<0.05 and ***, *P*<0.001.


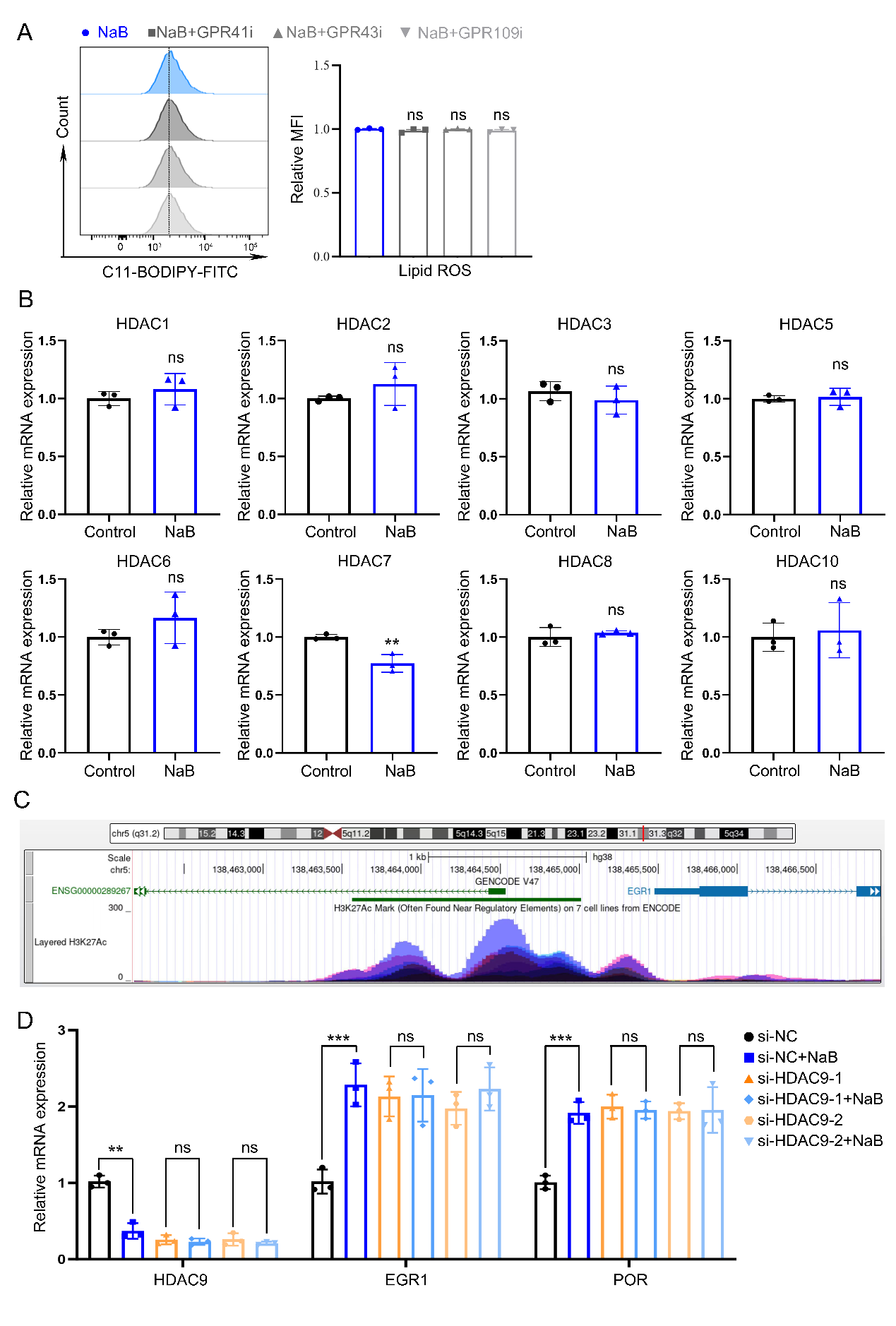


**Figure S4. NaB upregulates EGR1 via HDAC9 inhibition-mediated H3K27 acetylation.**

**(A)** Representative C11-BODIPY flow cytometry images, quantitative analysis of MFI in CAL27/CDDP treated with 5 mM NaB and combination with 5μM GPR41i, GPR43i and GPR109i for 24 hours.

**(B)** The other HDAC isoforms’ mRNA expression levels following NaB administration in CAl27/CDDP. **, *P*<0.05.

**(C)** The prediction of acetylation at H3K27 in the promoter region of EGR1.

**(D)** The mRNA expression levels of HDAC9, EGR1 and POR following HDAC9 knockdown and treated with 5 mM NaB in CAl27/CDDP. **, *P*<0.01 and ***, *P*<0.001.


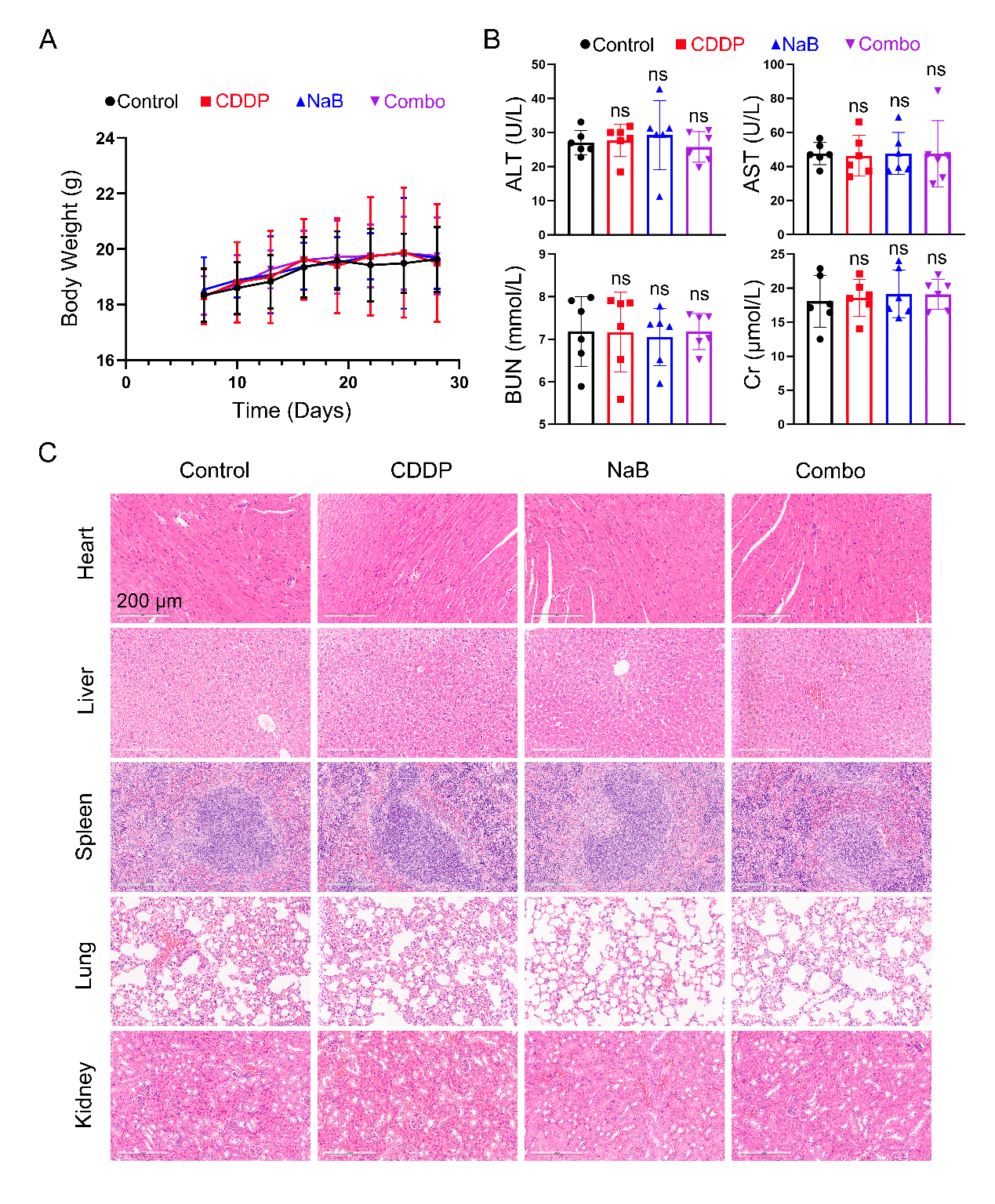


**Figure S5. Biosafety evaluation of NaB.**

**(A)** Line graph showing the body weight of nude mice with CDDP, NaB and their combined treatment.

**(B)** The level of ALT, AST, BUN and Cr for assessing the liver and kidney function in animals.

**(C)** Representative H&E images of animal organs. Scale bar, 200 μm.
