# Supplementary material for: Uncovering a novel treatment strategy: sodium butyrate overcomes cisplatin resistance in the oral squamous cell carcinoma by inducing ferroptosis
Source: J Exp Clin Cancer Res. 2026 Feb 16;45:66. doi: 10.1186/s13046-026-03663-0 (PMC12980862; doi:10.1186/s13046-026-03663-0)
Supplement: Supplementary file 2 — Supplementary Material 2. [file 13046_2026_3663_MOESM2_ESM.docx]

**Supplementary Table 1. Sequences of each primers** **used for qRT-PCR**

| **Names** | **5’ to 3’** | **Sequence** |
| --- | --- | --- |
| GPX4 | Forward primer  Reverse primer | TCGGCCGCCTTTGCC  CGATGTCCTTGGCGGAAAAC |
| FSP1 | Forward primer  Reverse primer | CGTCTACGCCATTGGTGACT  CACACCGTCATTTCTCCCCA |
| SLC7A11 | Forward primer  Reverse primer | ATGGTCAGAAAGCCTGTTGT  GCTCCAATGATGGTGCCAAT |
| SLC3A2 | Forward primer  Reverse primer | TGGAGCTACAGCCTCCTGAA  CCCGCGCTGAGACCCT |
| FPN1 | Forward primer  Reverse primer | GATCCTTGGCCGACTACCTG  TGCTGTCAAAAGGAGGCTGT |
| ACSL4 | Forward primer  Reverse primer | TGGTTCTACTGGCCGACCTA  TAGCACATGAGCCAAAGGCA |
| LAPCAT3 | Forward primer  Reverse primer | CCAGGAGCTGAGCCTTAACA  CAAAGCAAAGGGGTAACCCAG |
| ALOX15 | Forward primer  Reverse primer | GCCTAAGGCTGTGCTGAAGA  CACTGTTTTCCACCACGCTG |
| POR | Forward primer  Reverse primer | GTGGCACCCTTCATAGGCTT  CCCGGTACAGGTAGTCCTCA |
| TRFC | Forward primer  Reverse primer | TCGGAGAAACTGGACAGCAC  ATCACGCCAGACTTTGCTGA |
| HDAC1 | Forward primer  Reverse primer | CTCACCGAATCCGCATGACT  CTGCTTGCTGTACTCCGACA |
| HDAC2 | Forward primer  Reverse primer | ATGGCGTACAGTCAAGGAGG  TCATTTCTTCGGCAGTGGCT |
| HDAC3 | Forward primer  Reverse primer | ACCAGATCCGCCAGACAATC  AGTAGGCTGAAGTCCCTGCT |
| HDAC4 | Forward primer  Reverse primer | CGTCCGTTGGATGTCACAGA  CCTTCTCGTGCCACAAGTCT |
| HDAC5 | Forward primer  Reverse primer | CACACCCTGCTCTATGGGAC  ACAAGGCAGCACAGCATACA |
| HDAC6 | Forward primer  Reverse primer | ACCATCCAAGTCCATCGCAG  TGATGCCGAGGTGACTTTCC |
| HDAC7 | Forward primer  Reverse primer | CGGCATTCCCTACAGAACCC  CAGGTTGGGCTCAGAGACTG |
| HDAC8 | Forward primer  Reverse primer | TACTTGACCGGGGTCATCCT  CAGCTTGGCGTGATTTCCAG |
| HDAC9 | Forward primer  Reverse primer | TACTCAGGCGGAAGGATGGA  CCAGTTGGCCCATTGTTTGG |
| HDAC10 | Forward primer  Reverse primer | ATTTGACTCAGCCATCGGGG  CAGCAGCGTCTGTACTGTCA |
| EGR1 | Forward primer  Reverse primer | CACCTGACCGCAGAGTCTTT  CTGACCAAGCTGAAGAGGGG |
| CTCF | Forward primer  Reverse primer | GAATTGGTTCGGCATCGTCG  GTGTCCCTGCTGGCATAACT |
| FOSL2 | Forward primer  Reverse primer | ATCCCGGGAACTTTGACACC  TGGTGGGGATGAATGCACTG |
| SP1 | Forward primer  Reverse primer | GGGAAACGCTTCACACGTTC  ACCTGGGCCTCCCTTCTTAT |
| YY1 | Forward primer  Reverse primer | AGTGACCACCTTGCCAAACA  TTGCTGGTGGCGGAAGTATT |
| NR2F2 | Forward primer  Reverse primer | GGCCCGGGTAGCGACAA  GTCGATGGGACAGTTCCGGT |
| SREBF1 | Forward primer  Reverse primer | CTGACCGACATCGAAGGTGA  CCAGCATAGGGTGGGTCAAA |
| ZNF384 | Forward primer  Reverse primer | GGTAGCATCGACCCTAACCG  CATCCTCAGGGGAGAGGACA |
| HNF4A | Forward primer  Reverse primer | TGCGACTCTCCAAAACCCTC  ATTGCCCATCGTCAACACCT |
| β-actin | Forward primer  Reverse primer | TGTTGATGGAAAGCCACCGA  GTTTTCCATGCTGGCCTTGG |
| EGR1-Promoter | Forward primer  Reverse primer | GCTCTAGGCTTCCCCGAAG  GAGTGACGTGAAGCCCCC |
| GAPDH-Promoter | Forward primer  Reverse primer | TACTAGCGGTTTTACGGGCG  TCGAACAGGAGGAGCAGAAGAGCGA |
