# Supplementary material for: Uncovering a novel treatment strategy: sodium butyrate overcomes cisplatin resistance in the oral squamous cell carcinoma by inducing ferroptosis
Source: J Exp Clin Cancer Res. 2026 Feb 16;45:66. doi: 10.1186/s13046-026-03663-0 (PMC12980862; doi:10.1186/s13046-026-03663-0)
Supplement: Supplementary file 3 — Supplementary Material 3. [file 13046_2026_3663_MOESM3_ESM.docx]

**Supplementary Table 2. The list of antibodies used in this experiment.**

| **Name** | **Manufacturer** | **Product #** | **Application** | **Antigen retrieval buffer for IHC** | **References** |
| --- | --- | --- | --- | --- | --- |
| POR | Abcam | ab180597 | 1:1000 (WB); 1:1000 (IHC) | Tris/EDTA buffer (pH 9.0) | [1] |
| SLC3A2 | Immunoway | YT5599 | 1:1000 (WB) |  |  |
| SLC7A11 | Immunoway | YT8130 | 1:1000 (WB) |  |  |
| FSP1 | Proteintech | 20886 | 1:1000 (WB) |  |  |
| GPX4 | Abcam | ab125066 | 1:1000 (WB) |  |  |
| β-actin | Proteintech | 66009 | 1:5000 (WB) |  |  |
| 4-HNE | Bioss | Bs-6313R | 1:200(IHC) | sodium citrate buffer (pH 6.0) | [2] |
| HDAC9 | Abcam | Ab109446 | 1:1000 (WB); 1:1000 (IHC) | Tris/EDTA buffer (pH 9.0) | [3] |
| H3K27ac | Abcam | ab4729 | 1:1000 (WB) |  |  |
| EGR1 | Abcam | ab300449 | 1:1000 (WB); 1:500 (IHC) | Tris/EDTA buffer (pH 9.0) | [4] |
| HRP AffiniPure Goat Anti-Mouse IgG (H+L) | Emar | EM35110 | 1:10000 (WB) |  |  |
| HRP AffiniPure Goat Anti-Rabbit IgG (H+L) | Emar | EM35111 | 1:10000 (WB) |  |  |

1. Lidin E, Sköld MK, Angéria M, Davidsson J, Risling M. Hippocampal Expression of Cytochrome P450 1B1 in Penetrating Traumatic Brain Injury. International journal of molecular sciences. 2022;23(2).

2. Haberman Y, Tickle TL, Dexheimer PJ, Kim MO, Tang D, Karns R, Baldassano RN, Noe JD, Rosh J, Markowitz J, Heyman MB, Griffiths AM, Crandall WV, Mack DR, Baker SS, Huttenhower C, Keljo DJ, Hyams JS, Kugathasan S, Walters TD, Aronow B, Xavier RJ, Gevers D, Denson LA. Pediatric Crohn disease patients exhibit specific ileal transcriptome and microbiome signature. The Journal of clinical investigation. 2014;124(8):3617-3633.

3. Lei M, Lin H, Shi D, Hong P, Song H, Herman B, Liao Z, Yang C. Molecular mechanism and therapeutic potential of HDAC9 in intervertebral disc degeneration. Cellular & molecular biology letters. 2023;28(1):104.

4. Ghanem MA, Van der Kwast TH, Den Hollander JC, Sudaryo MK, Oomen MH, Noordzij MA, Van den Heuvel MM, Nassef SM, Nijman RM, Van Steenbrugge GJ. Expression and prognostic value of Wilms' tumor 1 and early growth response 1 proteins in nephroblastoma. Clinical cancer research : an official journal of the American Association for Cancer Research. 2000;6(11):4265-4271.
