# Supplementary material for: Uncovering a novel treatment strategy: sodium butyrate overcomes cisplatin resistance in the oral squamous cell carcinoma by inducing ferroptosis
Source: J Exp Clin Cancer Res. 2026 Feb 16;45:66. doi: 10.1186/s13046-026-03663-0 (PMC12980862; doi:10.1186/s13046-026-03663-0)
Supplement: Supplementary file 4 — Supplementary Material 4. [file 13046_2026_3663_MOESM4_ESM.docx]

**Supplementary Table 3. Sequences of each siRNA and shRNA**

| **siRNA** | **NO.** | **Target Sequence (5’ to 3’)** |
| --- | --- | --- |
| EGR1 | 1 | GCGGCAGAAGGACAAGAAATT  UUUCUUGUCCUUCUGCCGCTT |
|  | 2 | CCGACUACCUGUUUCCACATT  UGUGGAAACAGGUAGUCGGTT |
| HDAC9 | 1 | GCUGGUCAUUCAACAGCAATT  UUGCUGUUGAAUGACCAGCTT |
|  | 2 | GCAGAGGCAAGAACAGGAATT  UUCCUGUUCUUGCCUCUGCTT |
| **shRNA** | **NO.** | **Target Sequence (5’ to 3’)** |
| POR | 1 | ATCGTGGGTCTGACATTGACC |
|  | 2 | ACGAGCACTTCAATGCCATGG |
