# Supplementary material for: Uncovering a novel treatment strategy: sodium butyrate overcomes cisplatin resistance in the oral squamous cell carcinoma by inducing ferroptosis
Source: J Exp Clin Cancer Res. 2026 Feb 16;45:66. doi: 10.1186/s13046-026-03663-0 (PMC12980862; doi:10.1186/s13046-026-03663-0)
Supplement: Supplementary file 5 — Supplementary Material 5. [file 13046_2026_3663_MOESM5_ESM.docx]

**Supplementary Table 4. Sequences of POR promoter mutation**

| **Site** | **Sequence (5’ to 3’)** |
| --- | --- |
| WT | GGTACCGGTGACCTGCAGGGTCCGAGCTGTAGAAGCCGCAGCCGCCGTCTCCAGGCGACTCCGCCACCCCCGGAACCACGCACTTTCATTTCTCTGCCGGGCGACCCAGCCGAGCCGCGAGGGGGCGTGGCCGGGCCGGGCCGTACCAAGAGCGCAAATTTGCAGGGGGAAGCGCGGGGCTCCGGGAATGCACGCGCGGCTCGAGTGGGCGGGGCGGCGGCCCAATGGCCACGCCCCCACCCCCGCGGCCGGCGAAGGCGGTGGTAGCGCCTCAGTGGTGTGGGCCTGAGCCCTGCCCAGGTGCCCAAGCTT |
| MutA | GGTACCGGTGACCTGCAGGGTCCGAGCTGTAGAAGCCGCAGCCGCCGTCTCCAGGCGACTCCGCCACCCCCGGAACCACGCACTTTCATTTCTCTGCCGGGCGACCCAGCCGAGCCGCGAGGGGGCGTGGCCGGGCCGGGCCGTACCAAGAGCGCAAATTTGCAGGGGGAAGCGCGGGGCTCCGGGAATGCACGCGCGGCTCGAGTGGGCGGGGCGGCGGCCCAATGTAACATAAAAACAAAAATATGCCGGCGAAGGCGGTGGTAGCGCCTCAGTGGTGTGGGCCTGAGCCCTGCCCAGGTGCCCAAGCTT |
| MutB | GGTACCGGTGACCTGCAGGGTCCGAGCTGTAGAAGCCGCAGCCGCCGTCTCCAGGCGACTCCGCCACCCCCGGAACCACGCACTTTCATTTCTCTGCCGGGCGACCCAGCCGAGCCGCGAGGGGGCGTGGCCGGGCCGGGCCGTACCAAGAGCGCAAATTTGCAGGGGGAAGCGCGGGGCTCCGGGAATGCACGCGCGGCTCGAGTGGGCGGGGCGGCGGCCCAATGTAACATAAAAACAAAAATATGCCGGCGAAGGCGGTGGTAGCGCCTCAGTGGTGTGGGCCTGAGCCCTGCCCAGGTGCCCAAGCTT |
| MutC | GGTACCGGTGACCTGCAGGGTCCGAGCTGTAGAAGCCGCAGCCGCCGTCTCCAGGCGACTAATAACAAAAATTCACCACGCACTTTCATTTCTCTGCCGGGCGACCCAGCCGAGCCGCGAGGGGGCGTGGCCGGGCCGGGCCGTACCAAGAGCGCAAATTTGCAGGGGGAAGCGCGGGGCTCCGGGAATGCACGCGCGGCTCGAGTGGGCGGGGCGGCGGCCCAATGGCCACGCCCCCACCCCCGCGGCCGGCGAAGGCGGTGGTAGCGCCTCAGTGGTGTGGGCCTGAGCCCTGCCCAGGTGCCCAAGCTT |
